# Fulvestrant plus capivasertib versus placebo after relapse or progression on an aromatase inhibitor in metastatic, oestrogen receptor-positive, HER2-negative breast cancer (FAKTION): overall survival, updated progression-free survival, and expanded biomarker analysis from a randomised, phase 2 trial

**DOI:** 10.1016/S1470-2045(22)00284-4

**Published:** 2022-07

**Authors:** Sacha J Howell, Angela Casbard, Margherita Carucci, Kate Ingarfield, Rachel Butler, Sian Morgan, Magdalena Meissner, Catherine Bale, Pavel Bezecny, Sarah Moon, Chris Twelves, Ramachandran Venkitaraman, Simon Waters, Elza C de Bruin, Gaia Schiavon, Andrew Foxley, Robert H Jones

**Affiliations:** a The University of Manchester and The Christie NHS Foundation Trust, Manchester, UK; b Centre for Trials Research, Cardiff University, Cardiff, UK; c All Wales Laboratory Genetics Service, Cardiff, UK; d Cardiff and Vale University Health Board, Cardiff, UK; e Betsi Cadwaladr University Health Board, Bangor, UK; f Blackpool Teaching Hospitals NHS Foundation Trust, Blackpool, UK; g University Hospitals of Morecambe Bay NHS Foundation Trust, Lancaster, UK; h University of Leeds and Leeds Teaching Hospitals Trust, Leeds, UK; i The Ipswich Hospital NHS Trust, Ipswich, UK; j Velindre Cancer Centre, Cardiff, UK; k Oncology R&D, AstraZeneca, Cambridge, UK; l Cardiff University and Velindre Cancer Centre, Cardiff, UK

## Abstract

**Background:**

Capivasertib, an AKT inhibitor, added to fulvestrant, was previously reported to improve progression-free survival in women with aromatase inhibitor-resistant oestrogen receptor (ER)-positive, HER2-negative advanced breast cancer. The benefit appeared to be independent of the phosphoinositide 3-kinase (PI3K)/AKT/phosphatase and tensin homologue (PTEN) pathway alteration status of tumours, as ascertained using assays available at the time. Here, we report updated progression-free survival and overall survival results, and a prespecified examination of the effect of PI3K/AKT/PTEN pathway alterations identified by an expanded genetic testing panel on treatment outcomes.

**Methods:**

This randomised, multicentre, double-blind, placebo-controlled, phase 2 trial recruited postmenopausal adult women aged at least 18 years with ER-positive, HER2-negative, metastatic or locally advanced inoperable breast cancer and an Eastern Cooperative Oncology Group performance status of 0–2, who had relapsed or progressed on an aromatase inhibitor, from across 19 hospitals in the UK. Participants were randomly assigned (1:1) to receive intramuscular fulvestrant 500 mg (day 1) every 28 days (plus a 500 mg loading dose on day 15 of cycle 1) with either capivasertib 400 mg or matching placebo, orally twice daily on an intermittent weekly schedule of 4 days on and 3 days off, starting on cycle 1 day 15. Treatment continued until disease progression, unacceptable toxicity, loss to follow-up, or withdrawal of consent. Treatment was allocated by an interactive web-response system using a minimisation method (with a 20% random element) and the following minimisation factors: measurable or non-measurable disease, primary or secondary aromatase inhibitor resistance, *PIK3CA* status, and PTEN status. The primary endpoint was progression-free survival in the intention-to-treat population. Secondary endpoints shown in this Article were overall survival and safety in the intention-to-treat population, and the effect of tumour PI3K/AKT/PTEN pathway status identified by an expanded testing panel that included next-generation sequencing assays. Recruitment is complete. The trial is registered with ClinicalTrials.gov, number NCT01992952.

**Findings:**

Between March 16, 2015, and March 6, 2018, 183 participants were screened for eligibility and 140 (77%) were randomly assigned to receive fulvestrant plus capivasertib (n=69) or fulvestrant plus placebo (n=71). Median follow-up at the data cut-off of Nov 25, 2021, was 58·5 months (IQR 45·9–64·1) for participants treated with fulvestrant plus capivasertib and 62·3 months (IQR 62·1–70·3) for fulvestrant plus placebo. Updated median progression-free survival was 10·3 months (95% CI 5·0–13·4) in the group receiving fulvestrant plus capivasertib compared with 4·8 months (3·1–7·9) for fulvestrant plus placebo (adjusted hazard ratio [HR] 0·56 [95% CI 0·38–0·81]; two-sided p=0·0023). Median overall survival in the capivasertib versus placebo groups was 29·3 months (95% CI 23·7–39·0) versus 23·4 months (18·7–32·7; adjusted HR 0·66 [95% CI 0·45–0·97]; two-sided p=0·035). The expanded biomarker panel identified an expanded pathway-altered subgroup that contained 76 participants (54% of the intention-to-treat population). Median progression-free survival in the expanded pathway-altered subgroup for participants receiving capivasertib (n=39) was 12·8 months (95% CI 6·6–18·8) compared with 4·6 months (2·8–7·9) in the placebo group (n=37; adjusted HR 0·44 [95% CI 0·26–0·72]; two-sided p=0·0014). Median overall survival for the expanded pathway-altered subgroup receiving capivasertib was 38·9 months (95% CI 23·3–50·7) compared with 20·0 months (14·8–31·4) for those receiving placebo (adjusted HR 0·46 [95% CI 0·27–0·79]; two-sided p=0·0047). By contrast, there were no statistically significant differences in progression-free or overall survival in the expanded pathway non-altered subgroup treated with capivasertib (n=30) versus placebo (n=34). One additional serious adverse event (pneumonia) in the capivasertib group had occurred subsequent to the primary analysis. One death, due to atypical pulmonary infection, was assessed as possibly related to capivasertib treatment.

**Interpretation:**

Updated FAKTION data showed that capivasertib addition to fulvestrant extends the survival of participants with aromatase inhibitor-resistant ER-positive, HER2-negative advanced breast cancer. The expanded biomarker testing suggested that capivasertib predominantly benefits patients with PI3K/AKT/PTEN pathway-altered tumours. Phase 3 data are needed to substantiate the results, including in patients with previous CDK4/6 inhibitor exposure who were not included in the FAKTION trial.

**Funding:**

AstraZeneca and Cancer Research UK.

## Introduction

More than two-thirds of patients with advanced breast cancer have oestrogen receptor-positive (ER-positive), HER2-negative disease.^1^ Unless there is evidence of impending or actual visceral crisis, endocrine-based therapy is the preferred treatment modality because it has greater activity and better tolerability than cytotoxic chemotherapy.^2^ However, almost all tumours will become resistant to endocrine-based therapy and novel approaches are required to circumvent resistance, prolong the prechemotherapy window, and extend life.

The phosphoinositide 3-kinase (PI3K)/AKT/phosphatase and tensin homologue (PTEN) pathway, which promotes cell proliferation and resistance to apoptosis, is activated in several different types of cancer.^3^, ^4^ In ER-positive, HER2-negative breast cancers, PI3K/AKT/PTEN pathway activation occurs most frequently through *PIK3CA* mutations that activate the catalytic p110α subunit of PI3K.^5^, ^6^, ^7^, ^8^ Approximately 40% of advanced ER-positive tumours carry activating *PIK3CA* mutations. Additionally, a further 5–10% of advanced breast cancers harbour *AKT1*-activating mutations and 5–10% have inactivating alterations in *PTEN*.^6^, ^9^, ^10^, ^11^ Two agents that inhibit the PI3K/AKT/PTEN pathway have received regulatory approval for ER-positive, HER2-negative advanced breast cancer: the α-isoform-specific PI3K inhibitor, alpelisib, and the mTORC1 inhibitor, everolimus. Alpelisib given in combination with fulvestrant resulted in a significant progression-free survival benefit compared with fulvestrant alone, although only in participants with *PIK3CA*-mutant tumours, and it has not shown significantly prolonged overall survival compared with fulvestrant alone.^12^, ^13^ The addition of the mTORC1 inhibitor everolimus to either fulvestrant or an aromatase inhibitor has also been shown to significantly improve progression-free survival compared with endocrine therapy alone, although again no significant effect on overall survival has been established.^14^, ^15^

**Research in context**

**Evidence before this study**

We searched PubMed records published from Jan 1, 2009, to March 3, 2022, to identify publications directly relevant to the FAKTION clinical setting using the search terms “AKT” or “PI3K” or “mTOR” or “mTORC1” and “oestrogen receptor” and “breast cancer” and “metastatic” and “inhibitor” or “inhibition”. We also searched PubMed for publications in the same period using the terms “capivasertib” or “AZD5363”. We did not use any language restrictions. Before FAKTION, randomised studies had identified that pan-phosphoinositide 3-kinase (pan-PI3K) and β-sparing inhibitors had unfavourable toxicity profiles and low clinical activity, and these agents were no longer in development as breast cancer therapy. Two agents that inhibit the PI3K/AKT/PTEN pathway have received regulatory approval for patients with oestrogen receptor (ER)-positive, HER2-negative advanced breast cancer (alpelisib and everolimus), but neither have shown a significant prolongation of overall survival.

The primary analysis from FAKTION in 2020, was the first to report randomised data on the safety and efficacy of adding an AKT inhibitor to endocrine therapy for patients with advanced aromatase inhibitor-resistant ER-positive, HER2-negative breast cancer. To our knowledge, it also remains the only randomised study to do so. FAKTION showed that capivasertib plus fulvestrant significantly improved progression-free survival compared with fulvestrant plus placebo. Hazard ratios suggested that capivasertib had a similar benefit in subgroups that were defined as PI3K/AKT/PTEN pathway altered or pathway non-altered. Subsequent to the FAKTION publication, the phase 3 IPATunity130 trial found no progression-free survival benefit from adding the AKT inhibitor ipatasertib to paclitaxel in patients with PI3K/AKT/PTEN pathway-altered ER-positive, HER2-negative advanced breast cancer. This result aligned with that from the earlier phase 2 BEECH trial that tested capivasertib plus paclitaxel versus paclitaxel alone. By contrast, two single-group phase 1/2 studies showed that the combination of fulvestrant plus capivasertib resulted in objective response rates of 20–36% for advanced ER-positive, HER2-negative breast cancer that carried the activating *AKT1* E17K mutation, even after previous treatment with fulvestrant.

**Added value of this study**

To our knowledge, this follow-up analysis from FAKTION is the first demonstration that the addition of a PI3K/AKT/PTEN pathway inhibitor to endocrine therapy results in significantly longer overall survival in patients with aromatase inhibitor-resistant ER-positive, HER2-negative advanced breast cancer compared with endocrine therapy alone. Furthermore, expanded genetic testing identified *PIK3CA, AKT1*, and *PTEN* alterations in 25% of the tumours originally classified as pathway non-altered. The subsequent subgroup analysis suggested that capivasertib predominantly benefitted patients with PI3K/AKT/PTEN pathway-altered tumours.

**Implications of all the available evidence**

Although PI3K/AKT/PTEN pathway inhibitors alpelisib and everolimus are used in clinical practice as combination partners with endocrine-based therapy, they have not shown an overall survival benefit in patients whose ER-positive, HER2-negative advanced breast cancer progressed on an aromatase inhibitor. These updated results from FAKTION suggest that AKT inhibition with capivasertib might provide this benefit. The expanded biomarker results also suggest that genomic tumour profiling will be needed to accurately identify the approximately 50% of patients whose tumours carry relevant PI3K/AKT/PTEN pathway alterations and will most benefit from capivasertib.

AKT is the effector of the PI3K/AKT/PTEN pathway that is downstream of both PI3K and PTEN.^4^ Capivasertib is a potent and selective inhibitor of all three AKT isoforms (AKT1, AKT2, and AKT3).^16^ In the phase 2 FAKTION trial, we showed that the addition of capivasertib to fulvestrant endocrine therapy resulted in a significant improvement of progression-free survival in postmenopausal women with aromatase inhibitor-resistant ER-positive, HER2-negative advanced breast cancer.^17^ At the primary analysis, the median progression-free survival was 10·3 months (95% CI 5·0–13·2) in the fulvestrant plus capivasertib group versus 4·8 months (3·1–7·7) in the fulvestrant plus placebo group (hazard ratio [HR] 0·58 [95% CI 0·39–0·84]; p=0·0044). No safety concerns were identified and FAKTION results led to the design and initiation of the phase 3 CAPItello-291 trial (NCT04305496).

The FAKTION trial was designed in 2012, and had defined PI3K/AKT/PTEN pathway-altered status in terms of whether tumours carried one of four specific *PIK3CA* mutations (E542K or E545K in exon 9 or H1047R or H1047L in exon 20 detected by either pyrosequencing or digital-droplet PCR [ddPCR] tests (or both) on tumour tissue or cell-free DNA [cfDNA]), or displayed loss of PTEN expression by immunohistochemistry. *AKT1* E17K was not analysed. Using these original methods to identify tumour PI3K/AKT/PTEN pathway status, a secondary endpoint subgroup analysis suggested that the addition of capivasertib to fulvestrant conferred benefit for participants with either PI3K/AKT/PTEN pathway-altered advanced breast cancer or pathway non-altered advanced breast cancer (referred herein as the original pathway-altered and original pathway non-altered subgroups).^17^

It is becoming increasingly common to define tumour biomarker status with next-generation sequencing (NGS) technologies that can detect hundreds of alterations across multiple genes in a single test. A single NGS assay can sensitively detect activating *PIK3CA* mutations and *AKT1* mutations across their entire gene structures, as well as *PTEN* alterations and gene deletion. NGS testing has been previously used to identify PI3K/AKT/PTEN pathway-altered tumours.^18^, ^19^, ^20^

Here, we present the first mature analysis of overall survival and an updated progression-free survival analysis in the intent-to-treat population of FAKTION after an additional 34 months of follow-up. In a prespecified exploratory analysis, we considered the benefit of capivasertib by tumour PI3K/AKT/PTEN pathway-altered status after expanding testing of the originally collected tumour or plasma samples to include NGS assays. This testing identified an expanded pathway-altered subgroup of FAKTION participants whose tumours carried a *PIK3CA* mutation or *AKT1* E17K or deleterious *PTEN* alteration, as well as the corresponding expanded pathway non-altered subgroup.

## Methods

### Study design and participants

FAKTION was an investigator-initiated, multicentre, randomised, double-blind, placebo-controlled, biomarker-adaptive phase 2 trial that enrolled participants from 19 hospitals in the UK (appendix p 1).^17^ Eligible participants were postmenopausal women aged 18 years or older with an Eastern Cooperative Oncology Group performance status of 0–2 and local investigator-confirmed ER-positive, HER2-negative metastatic or locally advanced inoperable breast cancer (collectively termed advanced breast cancer) that was not amenable to curative surgical resection. ER-positive was defined as at least 10% of primary tumour or metastatic tumour cells staining positive for the oestrogen receptor. If no percentage score was available, a Quick (Allred) score of at least 4 of 8 was considered ER-positive. Participants were required to have histologically confirmed HER2-negative breast cancer from a biopsy collected at diagnosis or taken from a metastasis. Participants could have measurable or non-measurable disease according to Response Evaluation Criteria in Solid Tumours (RECIST) version 1.1. Participants' cancers were required to have relapsed on or within 12 months of adjuvant aromatase inhibitor therapy or have progressed on an aromatase inhibitor in the advanced setting (although this did not need to be their most recent therapy), with relapse or progression defined radiologically or by objective clinical evidence.

Participants were required to have a life expectancy of at least 12 weeks and adequate organ function (absolute neutrophil count ≥1·0 × 10^9^ per L; platelet count ≥100 × 10^9^ per L; haemoglobin ≥9 g/dL; international normalised ratio ≤1·5; potassium, calcium [corrected for serum albumin], and magnesium within normal limits for the institution; serum creatinine ≤1·5 times the upper limit of normal; alanine aminotransferase and aspartate aminotransferase ≤1·5 times the upper limit of normal [or <3·0 times if liver metastases]; total bilirubin ≤1·5 times the upper limit of normal; and fasting glucose <7·0 mmol/L). A full list of exclusion criteria have been previously published,^17^ including ineligibility if participants had clinically meaningful abnormalities of glucose metabolism, any evidence of severe or uncontrolled systemic diseases, or spinal cord compression or brain metastases unless asymptomatic, treated, and stable.

Up to three previous lines of endocrine therapy and one line of cytotoxic chemotherapy were permitted in the advanced setting. Individuals were ineligible if previously treated with fulvestrant or inhibitors of the PI3K/AKT/PTEN pathway, including an mTORC1 inhibitor. Participants were required to provide the most recent archival tumour sample and a baseline plasma sample. Written informed consent was obtained from all participants before trial screening procedures and enrolment. The trial was approved by the North West—Haydock Research Ethics Committee, Manchester, UK (13/NW/0842). The trial was done in accordance with the principles of Good Clinical Practice, the Declaration of Helsinki, and UK clinical trial regulations. The study protocol has been previously published.^17^

### Randomisation and masking

Participants were randomly assigned (1:1) to receive fulvestrant plus capivasertib or fulvestrant plus placebo. Randomisation was done centrally, using minimisation with a 20% random element.^21^ Minimisation factors were measurable versus non-measurable disease, primary versus secondary resistance to a third-generation aromatase inhibitor, and PI3K/AKT/PTEN pathway-altered status (as defined in the original analysis in which *PIK3CA* mutations were identified by pyrosequencing or ddPCR (or both), and PTEN deficiency was identified by immunohistochemistry). An interactive web-response system based on blinded drug pack number was used for treatment allocation. Participants were assigned six-digit trial numbers and treatment groups, and a confirmatory email including the participant's trial number, initials, date of birth, and treatment kit numbers was sent to the investigator. Capivasertib tablets and matching placebo had identical packaging, labelling, appearance, and administration schedules. Participants, investigators, study site staff, and sponsor were masked to treatment assignment until the database lock of the primary analysis.

### Procedures

Fulvestrant 500 mg was administered on day 1 of every 28-day cycle as two intramuscular injections, one into each buttock, and an additional loading dose (500 mg) was delivered on day 15 of cycle 1. Capivasertib 400 mg or matching placebo was given orally twice daily on an intermittent weekly schedule of 4 days on and 3 days off, starting on day 15 of cycle 1 (to facilitate the original biomarker testing before randomisation). Participants continued to receive study treatment until disease progression, development of unacceptable adverse events, loss to follow-up, or withdrawal of consent. Fulvestrant and capivasertib were manufactured and provided by AstraZeneca (Cambridge, UK) and distributed by Fisher Clinical Services (Horsham, UK). Adverse events suspected to be related to capivasertib were managed by dose interruption or dose reduction to 320 mg, then 240 mg, then 160 mg at the same schedule. Repeated dose interruptions and continuous interruption of up to 28 days were allowed. Participants were discontinued following a single dose interruption of more than 28 days. Dose reduction of fulvestrant to 250 mg was allowed after discussion with the chief investigators if an investigator felt that unacceptable toxicity could reasonably be attributed to fulvestrant or if there were physical difficulties with administration of bilateral injections.

CT scans of the chest, abdomen, and, if indicated, pelvis were done within 28 days before registration to confirm eligibility, and repeated every 8 weeks until cycle 7, then every 12 weeks until disease progression. Participants who discontinued study drugs for any reason other than progression continued to undergo imaging assessments until progression. Scans were assessed according to RECIST version 1.1 by local radiologists, without central review, to determine tumour response and date of progression.

Participant blood and tumour tissue samples were obtained after consent. They were centrally tested before randomisation at the All Wales Medical Genetics Service and the Department of Cellular Pathology, University Hospital of Wales, Cardiff, UK. The majority of tumour tissue samples had been collected from the primary tumour (97 [80%] of the 121 tissue samples). Plasma samples were collected from all participants during screening, before study treatment commenced. ddPCR or pyrosequencing (or both) of tissue or plasma samples were used to identify tumours that carried *PIK3CA* E542K, E545K, H1047L, or H1047R mutations, and immunohistochemistry was used to detect PTEN deficiency. These methods have been previously described in detail.^17^

After the original publication,^17^ we applied an existing ddPCR method^22^ to remaining tumour tissue samples to identify the activating *AKT1* E17K mutation. The FoundationOne CDx (F1CDx) NGS Clinical Trial Assay (Foundation Medicine, Cambridge, MA, USA) was used to detect single-nucleotide variations, insertion and deletion alterations, and copy number alterations in DNA isolated from remaining formalin-fixed paraffin-embedded tumour tissue specimens. The GuardantOMNI RUO (Guardant Health, Redwood City, CA, USA) detects single-nucleotide variations, insertion and deletion alterations, copy number alterations, or fusions in 500 genes, including *PIK3CA, AKT1*, and *PTEN* alterations using NGS of cfDNA extracted from remaining plasma samples.

A secondary statistical analysis plan that defined how participants would be assigned to expanded pathway-altered and pathway non-altered subgroups for an additional prespecified exploratory endpoint analysis was developed and signed off after the primary analysis but before the secondary database lock (appendix pp 10–37). Under the expanded testing, participant tumours were considered as PI3K/AKT/PTEN pathway altered if NGS testing of tumour tissue or plasma identified any of the following: *AKT1* E17K, *PIK3CA* R88Q, N345K, C420R, E542K, E545X, Q546X, M1043I, M1043V, H1047X, or G1049R (in which X represents any change in amino acid residue), a deleterious *PTEN* mutation, or loss of the *PTEN* gene. The expanded list of *PIK3CA* activating mutations corresponded to that previously used in the PAKT trial,^18^ plus the addition of M1043V based on its frequency in The Cancer Genome Atlas as well as in vitro and in vivo evidence that it was an activating *PIK3CA* mutation.^23^, ^24^ Participants were assigned to the expanded pathway-altered subgroup if they tested positive with any of the new assays, even if the tumour had originally been considered as pathway non-altered based on negative *PIK3CA* pyrosequencing or ddPCR and PTEN immunohistochemistry results.

We acquired a NGS testing result for 112 (80%) of 140 participants: 89 from a tissue sample, 68 from a plasma sample, and including 45 that had both tissue and plasma NGS results (appendix p 2). For the remainder (28 [20%] participants) for whom either no additional tissue or plasma sample was available, or the NGS assay failed, the statistical analysis plan included a prespecified algorithm to determine if a participant should be assigned to the expanded pathway-altered subgroup because their tumour carried a *PIK3CA* mutation or a *AKT1* mutation identified by ddPCR or pyrosequencing. This algorithm examined the concordance between ddPCR or pyrosequencing versus NGS detection of *PIK3CA* and *AKT1* mutations, and stated that if more than 90% of *PIK3CA* or *AKT1* mutations identified by ddPCR or pyrosequencing of tissue or plasma were also identified by NGS of tissue or plasma, then a positive tissue or plasma ddPCR or pyrosequencing result would be considered valid. The concordance between ddPCR or pyrosequencing (or both) and NGS testing was 86 (97%) of 89 for tissue samples and 68 (100%) of 68 for plasma samples (98% in total; appendix p 2). Thus, participants with tumours that had *PIK3CA* or *AKT1* mutations detected by ddPCR or pyrosequencing (or both) of tissue or plasma were included in the expanded pathway-altered subgroup when no NGS results were available. We did not assign participants to the expanded pathway-altered or pathway non-altered subgroups on the basis of results from the original PTEN assay.

The methods used to monitor FAKTION participants for adverse events have been previously described.^17^ In brief, participants were reviewed in clinic for adverse events and laboratory monitoring on day 1 of every cycle, at the end of treatment, and 30 days after treatment. Participants who discontinued treatment before progression were also monitored monthly. Blood was drawn for analysis of sodium, potassium, urea, creatinine, albumin, alanine transaminase or aspartate transaminase, alkaline phosphatase, bilirubin, calcium, and full blood count on day 1 of every cycle (and day 15 of cycle 1) to cycle 7 and every three cycles thereafter. Random blood glucose sampling followed the same pattern except that fasting blood glucose was measured instead of random glucose on cycle 1 day 15, cycle 2 day 1, and cycle 3 day 1. Participants took home urine glucose measurements on the third day of capivasertib or placebo dosing each week. Participants completed drug diaries, which were returned to the study site at each study visit to aid data collection.

Adverse events were classified according to the National Cancer Institute Common Terminology Criteria for Adverse Events (CTCAE) version 4.03. The incidence and severity of adverse events and serious adverse events were recorded throughout the study period, and haematological and biochemical laboratory tests were done every 4 weeks. The case report form prompted sites to review results for out-of-range laboratory test values and to report an adverse event by CTCAE grade if and when CTCAE criteria were met. Some adverse events identified from abnormal blood or biochemistry laboratory testing results might not have had clinical significance. Serious adverse events could be reported at any time.

### Outcomes

The primary endpoint was investigator-assessed progression-free survival, defined as the time from randomisation to either the first documented progression confirmed by RECIST version 1.1 criteria (regardless of whether the participant withdrew from the study or received another anticancer therapy before progression) or death from any cause. Secondary endpoints with updated data were the effect of *PIK3CA* mutational status and PTEN expression on progression-free survival and overall survival (the original testing panel), overall survival (defined as the time from randomisation to death from any cause), exploratory biomarkers, and safety. The effect of capivasertib on fulvestrant pharmacokinetics, the tolerability of capivasertib plus fulvestrant, objective response (defined as the proportion of participants with a complete or partial response, according to RECIST version 1.1), and clinical benefit (defined as the proportion of participants with an objective response or stable disease lasting ≥24 weeks) were additional prespecified secondary outcomes for which no new analyses were done at this update.

### Statistical analysis

The FAKTION study protocol and statistical analysis has been previously published^17^ and the same methods were used in this updated analysis. In brief, the primary hypothesis was that participants treated with fulvestrant plus capivasertib would have improved median progression-free survival compared with those treated with fulvestrant plus placebo. The sample size was calculated for a phase 2 screening design, based on a primary outcome of progression-free survival, assuming a time-to-event hazard ratio of 0·65, 90% power, a one-sided α of 0·20, and an overall loss to follow-up of 10%. A total of 98 events were required in 138 participants with 18-month accrual and 6-month minimum follow-up.

The primary and secondary analyses were performed in the full analysis set, comprising all randomly assigned participants, on an intention-to-treat basis. An interim analysis of change in tumour size 8 weeks after randomisation in the first 40 participants without pathway alteration was planned to allow adaptation of recruitment according to participants' pathway alteration status and has been previously published.^17^ Time-to-event distributions were estimated with the Kaplan-Meier method. The significance threshold was 0·05. Participants with no follow-up RECIST assessment were censored at day 1 unless they died within two visits of baseline (16 weeks plus 1 week allowing for a late assessment within the visit window), in which case they were censored at their death date. Participants without disease progression confirmed by RECIST and those who died or progressed after missing the last two RECIST assessments were censored for progression-free survival at the date of the last evaluable RECIST assessment or at the point of withdrawal of consent. Cox regression was used to estimate HRs with CIs and p values; multivariable Cox regression was used to adjust the estimates for the randomisation minimisation variables. HRs were adjusted for pathway status as determined at randomisation, primary or secondary aromatase inhibitor resistance, and measurable or non-measurable disease. This adjustment was prespecified in the original statistical analysis plan and the secondary statistical analysis plan that applied to this analysis. Overall survival was measured from randomisation to death, with participants still alive censored at the date last assessed. The overall survival data were summarised and analysed in the same way as progression-free survival. The proportional hazards assumption was checked using Cox-Snell residuals and Schoenfeld's global test (appendix p 3).

This follow-up analysis took place with a data cutoff of Nov 25, 2021. The secondary statistical analysis plan (appendix pp 10–37; developed and approved before the data cutoff) specified that overall survival would be analysed after 98 deaths had occurred in the intention-to-treat population. It also defined the prespecified exploratory endpoint of analysing progression-free survival and overall survival in subgroups in which the identification of PI3K/AKT/PTEN pathway-alteration status versus pathway non-alteration status was expanded to include *AKT1* testing (expanded testing panel) and results from NGS assays. As for the original secondary endpoint, the hypothesis was that the combination of fulvestrant and capivasertib would show greater benefit in participants whose tumours carried PI3K/AKT/PTEN pathway alterations, as examined by analysis of progression-free survival and overall survival outcomes in expanded pathway-altered and non-altered subgroups, using the same statistical tests as described for the intention-to-treat population. There was no adjustment for multiplicity of testing. Two-sided p values were reported (p≤0·05 was considered significant). Schoenfeld's tests for overall survival and progression-free survival in the intention-to-treat population, the expanded pathway-altered, and the expanded pathway non-altered subgroups were consistent with the proportional hazards assumption, and the assumption was thus adequately met (appendix p 3). The exploratory endpoints examining the benefit of fulvestrant plus capivasertib versus fulvestrant plus placebo in the pathway-altered and pathway non-altered subgroups identified by NGS alone were defined post hoc and were not prespecified. All statistical analyses were done using Stata (version 17.0). This trial is registered with ClinicalTrials.gov (NCT01992952).

### Role of the funding source

The funder (AstraZeneca) supplied capivasertib, matching placebo, and fulvestrant, contributed to the study design, reviewed the draft analysis plan, and provided critical review of the draft report, including interpretation, but had no role in data collection or data analysis. The co-funder (Cancer Research UK) approved the study design but had no role in the drafting of the report, or data collection, analysis, or interpretation.

## Results

**Figure 1:**
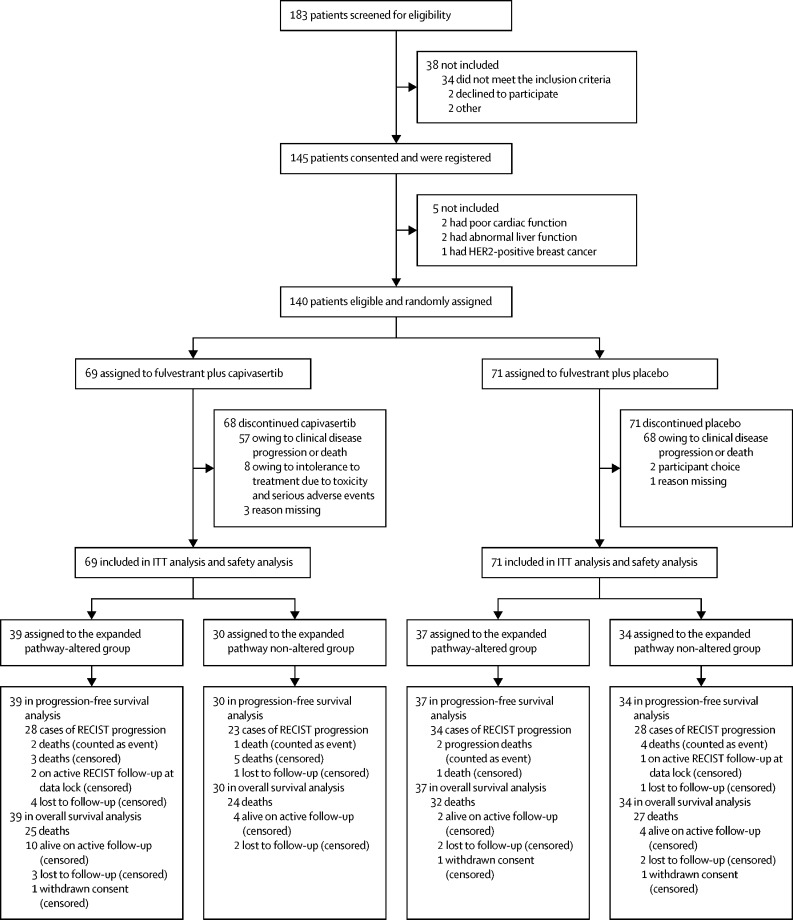
Trial profile ITT=intention-to-treat. RECIST=Response Evaluation Criteria in Solid Tumors.

From March 16, 2015, to March 6, 2018, 183 participants were screened for eligibility and 140 were randomly assigned to receive fulvestrant plus capivasertib (n=69 [49%]) or fulvestrant plus placebo (n=71 [51%]; figure 1). Baseline participant characteristics were reported for the primary analysis^17^ and are presented in the appendix (p 4); all participants were women and FAKTION did not collect data on race or ethnicity.

**Figure 2:**
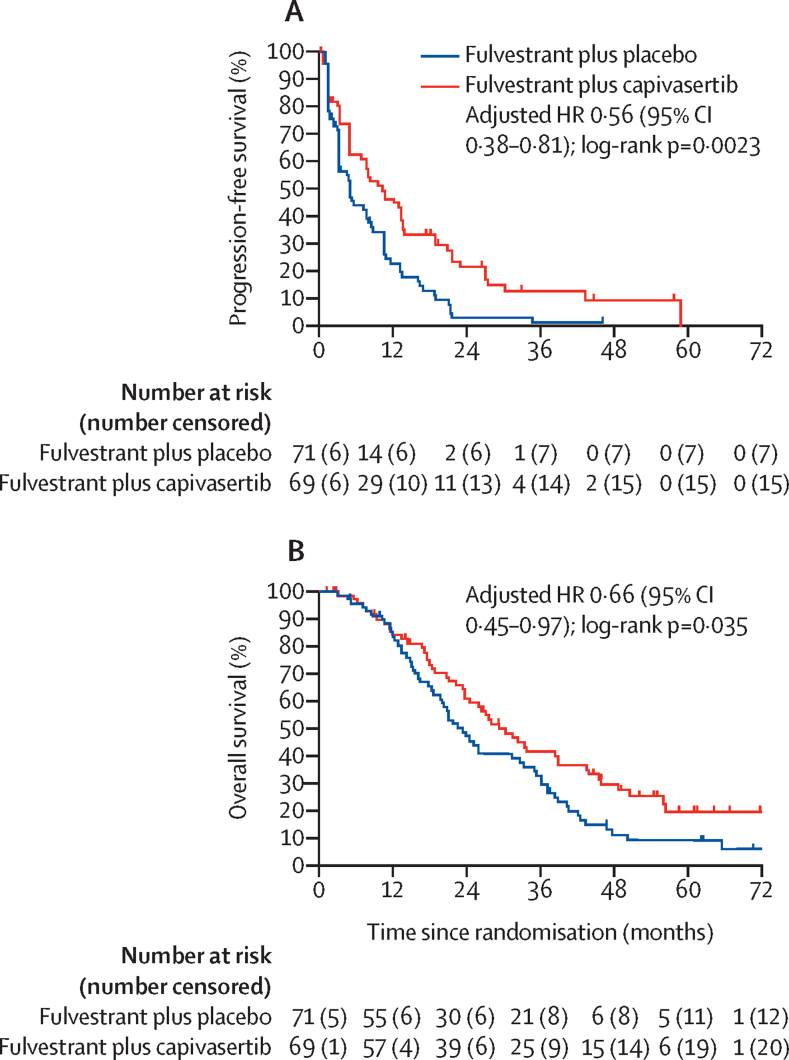
Progression-free survival (A) and overall survival (B) in the intention-to-treat population Tick marks on plots show censoring events. HR=hazard ratio.

Median follow-up at the data cutoff of Nov 25, 2021, was 58·5 months (IQR 45·9–64·1) for participants treated with fulvestrant plus capivasertib and 62·3 months (62·1–70·3) with fulvestrant plus placebo. The progression-free survival analysis in the intention-to-treat population was updated after 118 progression events had occurred (54 [78%] of the 69 participants assigned to capivasertib and 64 [90%] of the 71 participants assigned to placebo), and a significant benefit for the addition of capivasertib continued to be shown. The fulvestrant plus capivasertib group had a median progression-free survival of 10·3 months (95% CI 5·0–13·4) and the fulvestrant plus placebo group had a median progression-free survival of 4·8 months (3·1–7·9; unadjusted HR 0·55 [95% CI 0·38–0·80]; two-sided p=0·0019; adjusted HR 0·56 [95% CI 0·38–0·81]; two-sided p=0·0023; table 1; figure 2A).

**Table 1:** Progression-free survival and overall survival in the intention-to-treat population and the PI3K/AKT/PTEN pathway-altered and pathway non-altered subgroups identified by original, expanded, and NGS testing

		**Fulvestrant plus capivasertib group**		**Fulvestrant plus placebo group**		**Unadjusted HR (95% CI); two-sided p value**	**Adjusted HR (95% CI); two-sided p value**
		Number of events/number of patients (%)	Median (95% CI), months	Number of events/number of patients (%)	Median (95% CI), months		
Progression-free survival in intention-to-treat population, months		54/69 (78%)	10·3 (5·0–13·4)	64/71 (90%)	4·8 (3·1–7·9)	0·55 (0·38–0·80); p=0·0019	0·56 (0·38–0·81); p=0·0023
Overall survival in intention-to-treat population, months		49/69 (71%)	29·3 (23·7–39·0)	59/71 (83%)	23·4 (18·7–32·7)	0·66 (0·45–0·96); p=0·030	0·66 (0·45–0·97); p=0·035
Progression-free survival in pathway-altered subgroups							
	Original pathway-altered subgroup	26/31 (83%)	10·5 (6·6–18·7)	28/28 (100%)	5·2 (3·1–8·4)	0·51 (0·30–0·89); p=0·018	0·47 (0·26–0·84); p=0·011
	Expanded pathway-altered subgroup	30/39 (77%)	12·8 (6·6–18·8)	36/37 (97%)	4·6 (2·8–7·9)	0·46 (0·28–0·75); p=0·0021	0·44 (0·26–0·72); p=0·0014
	NGS-identified pathway-altered subgroup	25/34 (74%)	13·4 (6·6–20·7)	29/29 (100%)	3·1 (2·8–7·7)	0·35 (0·20–0·63); p=0·0004	0·36 (0·20–0·65); p=0·0007
Progression-free survival in pathway non-altered subgroups							
	Original pathway non-altered subgroup	28/38 (74%)	10·3 (3·2–13·5)	36/43 (84%)	4·8 (3·0–10·3)	0·59 (0·35–0·99); p=0·044	0·59 (0·35–0·98); p=0·042
	Expanded pathway non-altered subgroup	24/30 (80%)	7·7 (3·1–13·2)	28/34 (82%)	4·9 (3·2–10·5)	0·72 (0·41–1·27); p=0·25	0·70 (0·40–1·25); p=0·23
	NGS-identified pathway non-altered subgroup	18/22 (82%)	4·8 (1·3–10·3)	22/27 (81%)	5·2 (2·2–10·5)	0·95 (0·50–1·81); p=0·88	0·95 (0·49–1·82); p=0·87
Overall survival in pathway-altered subgroups							
	Original pathway-altered subgroup	20/31 (64%)	33·5 (22·3–50·7)	24/28 (86%)	20·9 (15·5–36·1)	0·51 (0·28–0·94); p=0·030	0·50 (0·27–0·92); p=0·025
	Expanded pathway-altered subgroup	25/39 (64%)	38·9 (23·3–50·7)	32/37 (86%)	20·0 (14·8–31·4)	0·49 (0·29–0·84); p=0·0091	0·46 (0·27–0·79); p=0·0047
	NGS-identified pathway-altered subgroup	21/34 (61%)	39·0 (22·3–50·7)	25/29 (86%)	20·9 (14·1–35·4)	0·43 (0·24–0·78); p=0·0056	0·44 (0·24–0·81); p=0·0076
Overall survival in pathway non-altered subgroups							
	Original pathway non-altered subgroup	29/38 (76%)	26·2 (20·7–38·5)	35/43 (81%)	23·9 (16·3–33·3)	0·80 (0·47–1·30); p=0·37	0·80 (0·49–1·32); p=0·39
	Expanded pathway non-altered subgroup	24/30 (80%)	26·0 (18·4–33·8)	27/34 (79%)	25·2 (20·3–36·2)	0·95 (0·55–1·64); p=0·85	0·86 (0·49–1·52); p=0·60
	NGS-identified pathway non-altered subgroup	17/22 (77%)	23·7 (16·7–38·5)	22/27 (81%)	25·2 (15·3–38·8)	0·87 (0·46–1·64); p=0·66	0·86 (0·45–1·63); p=0·64

HR=hazard ratio. NGS=next-generation sequencing. NR=not reached.

108 deaths had occurred in the intention-to-treat population: 49 (71%) of the 69 participants assigned capivasertib and 59 (83%) of the 71 participants assigned placebo. Median overall survival was 29·3 months (95% CI 23·7–39·0) for the fulvestrant plus capivasertib group and 23·4 months (18·7–32·7) for the fulvestrant plus placebo group (unadjusted HR 0·66 [0·45–0·96]; two-sided p=0·030; adjusted HR 0·66 [95% CI 0·45–0·97]; two-sided p=0·035; table 1; figure 2B).

An updated analysis of toxicity and safety data was done (appendix pp 5–6). There was relatively little change in the frequency of adverse events from the primary analysis.^17^ The most common grade 3 or 4 adverse events were hypertension (22 [32%] of 69 participants in the fulvestrant plus capivasertib group *vs* 18 [25%] of 71 participants in the fulvestrant plus placebo group), diarrhoea (ten [14%] *vs* three [4%]), and rash (14 [20%] *vs* 0). There was one additional serious adverse event (pneumonia) reported in the fulvestrant plus capivasertib group since the data cutoff of the primary analysis^17^ that was suspected to have a causal relationship with treatment. A death from an atypical pulmonary infection (without disease progression) occurred in the group treated with capivasertib and was considered as possibly related to treatment. One death in the capivasertib group (due to a thrombotic cerebrovascular accident) occurred a year after the end of treatment. Four deaths in the capivasertib treatment group and three in the placebo group were of unknown cause. All remaining deaths in both groups (43 in the capivasertib group and 56 in the placebo group) were disease related.

**Figure 3:**
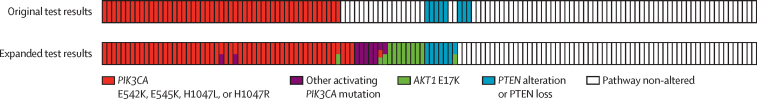
Participants with PI3K/AKT/PTEN pathway-altered tumours identified by the expanded biomarker testing and compared with the original testing results Multiple colours indicate that a participant tumour carried two or more types of pathway alteration.

**Figure 4:**
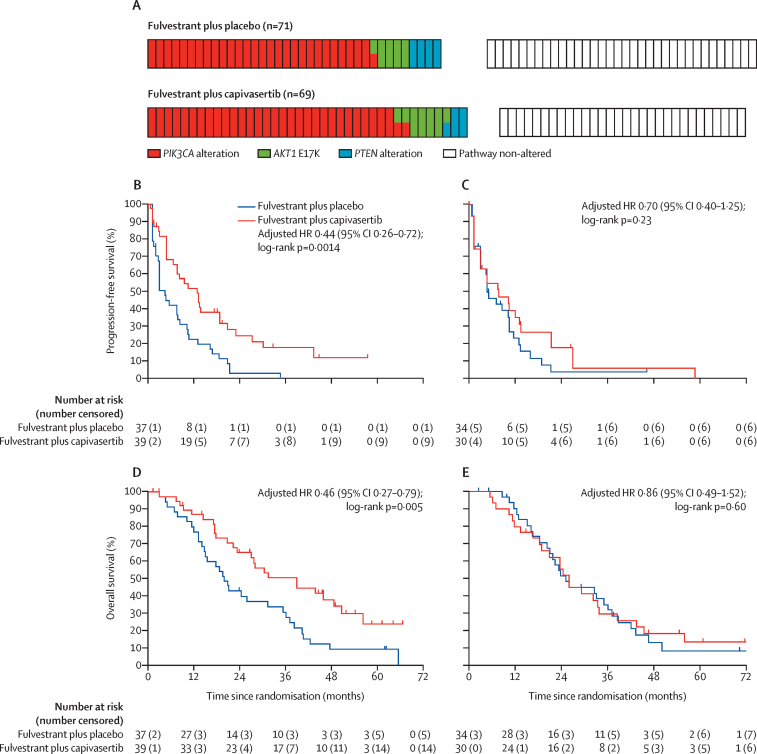
Progression-free survival and overall survival in the expanded PI3K/AKT/PTEN pathway-altered subgroup and pathway non-altered subgroup Tick marks on plots show censoring events. (A) Genetic profile of tumours in each group as identified by expanded biomarker testing; two colours indicate that a tumour carried mutations in both genes. (B) Progression-free survival in the expanded pathway-altered subgroup. (C) Progression-free survival in the expanded pathway non-altered subgroup. (D) Overall survival in the expanded pathway-altered subgroup. (E) Overall survival in the expanded pathway non-altered subgroup. HR=hazard ratio.

In the expanded testing panel that used advances in genetic testing assays, *PIK3CA, AKT1* and *PTEN* alterations were identified in tumours from 76 (54%) of the 140 participants in the intention-to-treat population (39 who received capivasertib and 37 who received placebo). Tumour alterations were identified in 20 (25%) of the 81 participants whose tumours had been originally considered as pathway non-altered (ten of whom had been assigned capivasertib and ten placebo; figure 3; appendix p 7). The treatment assignment of the pathway-altered and pathway non-altered subgroups remained balanced, including for the proportion of participants who had received two or more lines of previous therapy in the metastatic setting (table 2). The treatment groups were also balanced for the proportion of participants whose tumours carried *PIK3CA* mutations versus *AKT1* mutations versus *PTEN* alterations (figure 4A).

**Table 2:** Baseline characteristics of patients in the expanded PI3K/AKT/PTEN pathway-altered and pathway non-altered subgroups

		**Expanded pathway-altered subgroup**		**Expanded pathway non-altered subgroup**	
		Fulvestrant plus capivasertib (n=39)	Fulvestrant plus placebo (n=37)	Fulvestrant plus capivasertib (n=30)	Fulvestrant plus placebo (n=34)
Median age, years (IQR); range		60 (55–69); 46–81	62 (56–68); 47–73	62 (57–68); 42–79	60 (52–67); 40–82
ECOG performance status (physical examination)					
	0	25 (64%)	25 (68%)	17 (57%)	24 (71%)
	1	14 (36%)	9 (24%)	11 (37%)	8 (24%)
	2	0	1 (3%)	1 (3%)	1 (3%)
	Missing data	0	2 (5%)	1 (3%)	1 (3%)
Histopathological subtype					
	Invasive ductal carcinoma	33 (85%)	31 (84%)	24 (80%)	27 (79%)
	Invasive lobular cancer	2 (5%)	5 (14%)	2 (7%)	7 (21%)
	Mixed invasive ductal carcinoma and invasive lobular cancer	3 (8%)	0	2 (7%)	0
	Other	1 (3%)	1 (3%)	2 (7%)	0
Stage					
	III inoperable	0	1 (3%)	0	0
	IV	38 (97%)	35 (95%)	30 (100%)	33 (97%)
	Missing	1 (3%)	1 (3%)	0	1 (3%)
Number of disease sites					
	Median (IQR); range	2 (2–3); 1–5	2 (1–3); 1–5	2 (2–3); 1–5	2 (2–3); 1–5
	1	8 (21%)	11 (30%)	5 (17%)	8 (24%)
	2	31 (79%)	26 (70%)	25 (83%)	26 (76%)
Metastatic sites*					
	Brain	1 (3%)	1 (3%)	0	0
	Liver	22 (56%)	12 (32%)	10 (33%)	17 (50%)
	Lung	17 (44%)	17 (46%)	13 (43%)	11 (32%)
	Bone	34 (87%)	28 (76%)	25 (83%)	27 (79%)
	Lymph	14 (36%)	19 (51%)	14 (47%)	12 (35%)
	Pericardial or pleural	2 (5%)	0	3 (10%)	3 (9%)
	Chest wall or skin	0	2 (5%)	1 (3%)	1 (3%)
	Other visceral	2 (5%)	0	0	0
Visceral disease		30 (77%)	24 (65%)	19 (63%)	23 (68%)
Measurable disease†		27 (69%)	26 (70%)	22 (73%)	24 (71%)
Primary or secondary aromatase inhibitor resistance†					
	Primary	15 (38%)	10 (27%)	10 (33%)	16 (47%)
	Secondary	24 (62%)	27 (73%)	20 (67%)	18 (53%)
Previous breast surgery		34 (87%)	32 (86%)	25 (83%)	30 (88%)
Previous adjuvant endocrine therapy		34 (87%)	35 (95%)	26 (87%)	30 (88%)
	Any tamoxifen	23 (59%)	23 (62%)	18 (60%)	22 (65%)
	Any aromatase inhibitor	22 (56%)	21 (57%)	18 (60%)	17 (50%)
	Any gonadotropin-releasing hormone	2 (5%)	0	1 (3%)	1 (3%)
	Other	1 (3%)	0	0	1 (3%)
	Missing	0	0	0	1 (3%)
Previous adjuvant chemotherapy		20 (51%)	21 (57%)	16 (53%)	21 (62%)
	Anthracycline based	7 (18%)	10 (27%)	4 (13%)	3 (9%)
	Taxane based	1 (3%)	4 (11%)	4 (13%)	1 (3%)
	Anthracycline plus taxane	8 (21%)	2 (5%)	3 (10%)	7 (21%)
	Cyclophosphamide, methotrexate, and fluorouracil or capecitabine	2 (5%)	4 (11%)	5 (17%)	10 (29%)
	Other	1 (3%)	1 (3%)	0	0
	Missing	1 (3%)	0	0	0
Previous endocrine treatment (metastatic or locally advanced setting)					
	0 lines	6 (15%)	2 (5%)	3 (10%)	4 (12%)
	1 line	22 (56%)	26 (70%)	17 (57%)	19 (56%)
	≥2 lines	11 (28%)	9 (24%)	9 (30%)	11 (32%)
	Missing	0	0	1 (3%)	0
Metastatic chemotherapy for advanced breast cancer		9 (23%)	9 (24%)	8 (27%)	11 (32%)
	Capecitabine based	0	1 (3%)	3 (10%)	5 (15%)
	Taxane based	5 (13%)	5 (14%)	3 (10%)	3 (9%)
	Anthracycline based	2 (5%)	3 (8%)	0	3 (9%)
	Combined anthracycline and taxane	1 (3%)	0	2 (7%)	0
	Other	1 (3%)	0	0	0

Data are n (%) unless otherwise stated. The displayed percentages include missing values. ECOG=Eastern Cooperative Oncology Group.

* Sites are not mutually exclusive.

† Randomisation minimisation factor.

At the time of analysis, median follow-up in the expanded pathway-altered subgroup was 54·3 months (IQR 45·5–61·2) for the fulvestrant and capivasertib group and 62·3 months (62·1–not reached) for the fulvestrant and placebo group. For the 64 participants in the expanded pathway non-altered subgroup, median follow-up was 60·9 months (IQR 54·8–71·6) for those receiving fulvestrant and capivasertib and 70·3 months (46·6–74·2) for those receiving fulvestrant and placebo. A progression-free survival event was recorded for 66 (87%) of 76 participants in the expanded pathway-altered subgroup: 30 (77%) of 39 participants who received capivasertib and 36 (97%) of 37 participants who received placebo. Progression events had been recorded for 52 (81%) of the 64 participants in the expanded pathway non-altered group: 24 (80%) of 30 participants who received capivasertib and 28 (82%) of 34 participants who received placebo. Median progression-free survival was longer in the expanded pathway-altered subgroup who received fulvestrant plus capivasertib than in those who received fulvestrant plus placebo: 12·8 months (95% CI 6·6–18·8) in the fulvestrant plus capivasertib group versus 4·6 months (2·8–7·9) in the fulvestrant plus placebo group (adjusted HR 0·44 [95% CI 0·26–0·72]; two-sided p=0·0014; table 1; figure 4B). For the expanded pathway non-altered subgroup, median progression-free survival was 7·7 months (95% CI 3·1–13·2) for participants who received capivasertib and 4·9 months (3·2–10·5) for participants who received placebo (adjusted HR 0·70 [95% CI 0·40–1·25], p=0·23; table 1; figure 4C).

At the time of analysis, 57 (75%) of 76 participants in the expanded pathway-altered subgroup had died: 25 (64%) of the 39 participants who received capivasertib and 32 (86%) of the 37 participants who received placebo. 51 (80%) of 64 participants in the expanded pathway non-altered subgroup had died: 24 (80%) of the 30 participants who received capivasertib and 27 (79%) of the 34 participants who received placebo. Median overall survival in the expanded pathway-altered subgroup receiving fulvestrant plus capivasertib was statistically significantly longer than in the subgroup receiving fulvestrant plus placebo (table 1; figure 4D). In contrast, overall survival was similar in the two treatment groups of the expanded pathway non-altered subgroup (table 1; figure 4E). We also updated progression-free survival and overall survival analyses for the original pathway-altered subgroup and pathway non-altered subgroup identified by *PIK3CA* ddPCR or pyrosequencing (or both) and PTEN immunohistochemistry. The addition of capivasertib showed a significant progression-free survival benefit in the original pathway-altered subgroup and, as seen in the primary analysis,^17^ in the original pathway non-altered subgroup (table 1).

NGS and the original ddPCR or pyrosequencing results were acquired for tumours from 56 (79%) of the 71 participants treated with placebo and 56 (81%) of the 69 participants who received capivasertib (112 [80%] of the 140 patients in the intention-to-treat population). Of the 28 (20%) participants in the intention-to-treat population who did not have an additional tissue or plasma sample available, or for whom the NGS assay failed, 13 (19%) were in the subgroup of 69 treated with capivasertib and 15 (21%) were in the subgroup of 71 who received placebo. NGS identified several *PIK3CA* mutations, *AKT1*, or *PTEN* loss-of-function alterations that were not reported by the original tests (appendix p 2). By implication, this finding raised the possibility that the 20 participants who did not have NGS results, and were considered as pathway non-altered in the expanded analysis on the basis of negative original genetic test results, might in fact have had PI3K/AKT/PTEN pathway-altered tumours; these participants amounted to 20 (31%) of 64 of the expanded pathway non-altered subgroup (ten had received capivasertib and ten had received placebo). To account for these findings, we did a post-hoc analysis of efficacy data from participants for whom NGS testing of a tissue or plasma sample determined pathway-altered status. Of the 112 participants with NGS results, 63 (56%) had pathway altered tumours (34 had received capivasertib and 29 had received placebo). The groups remained relatively balanced, although the pathway-altered subgroup assigned to receive capivasertib contained more participants with *AKT1* E17K (seven [20%] of 34) than the subgroup assigned placebo (two [7%] of 29; appendix p 9). A progression-free survival event had been recorded for 54 (86%) of the 63 participants in the NGS-identified pathway-altered subgroup (34 who had received capivasertib and 29 who had received placebo) and 40 (82%) of 49 participants in NGS-identified pathway non-altered subgroup (22 who had received capivasertib and 27 who had received placebo). In the NGS-identified pathway-altered subgroup, progression-free survival was longer with capivasertib than with placebo (table 1; appendix p 9). In the NGS-identified pathway non-altered subgroup, progression-free survival was similar for both treatment groups (appendix p 9). Overall survival results are shown in appendix (p 9) and table 1.

## Discussion

This updated analysis of the phase 2 FAKTION trial after 5 years of follow-up showed that the addition of capivasertib to fulvestrant resulted in a clinically and statistically significant prolongation of overall survival in participants with aromatase inhibitor-resistant ER-positive, HER2negative advanced breast cancer. Additionally, exploratory subgroup analyses highlighted the value of using NGS to identify tumour mutational status and suggested that capivasertib predominantly benefited participants with PI3K/AKT/PTEN pathway-altered tumours.

Deregulation of the PI3K/AKT/PTEN signalling pathway is associated with the development and maintenance of many tumours. Here, we detected activating *PIK3CA* mutations, *AKT1* mutations, and inactivating *PTEN* alterations in 76 (54%) of an intention-to-treat population of postmenopausal women with ER-positive, HER2-negative advanced breast cancer who had previously been treated with an aromatase inhibitor. This high frequency is similar to that reported by comprehensive genomic profiling.^7^, ^9^, ^11^ Neither of the two currently approved drugs that inhibit the PI3K/AKT/PTEN pathway (the PI3K inhibitor alpelisib or mTORC1 inhibitor everolimus) added to endocrine therapy have been shown to significantly extend overall survival compared with endocrine therapy alone,^13^, ^15^ highlighting the need for new breast cancer therapies against the pathway. The mature overall survival data reported here show that capivasertib has potential to fill this treatment gap.

Although other trials have examined AKT inhibition in combination with chemotherapy for breast cancer, FAKTION is, to our knowledge, the only randomised trial reporting data from combining an AKT inhibitor with endocrine therapy to date. Given the limitations of the trial, future research will be important to support the results from FAKTION. The overall survival result should be assessed in a larger cohort and information on subsequent therapies that was not available for participants in FAKTION should be ascertained. FAKTION was also a phase 2 screening study, with a relaxed type 1 error and one-sided design that had the goal of detecting an active drug, but with an increased risk of obtaining a false-positive result. Moreover, FAKTION participants were recruited between 2015 and 2018, and had no exposure to the cyclin-dependent kinase 4/6 (CDK4/6) inhibitors that are now first-line standard of care in combination with endocrine therapy in this setting. Although the results from FAKTION do not guarantee that capivasertib will benefit patients who progressed on combined CDK4/6 inhibitor and endocrine therapy, existing clinical data suggest that previous exposure to a CDK4/6 inhibitor does not abrogate the efficacy of therapies that inhibit the PI3K/AKT/PTEN pathway.^25^, ^26^, ^27^ Moreover, there is evidence that PI3K/AKT/PTEN pathway deregulation is key to the development of resistance to CDK4/6 inhibitors,^28^, ^29^ which further supports the importance of testing whether capivasertib benefits patients who have received one of these therapies. The ongoing phase 3 CAPItello-291 trial (NCT04305496) is assessing the efficacy and safety of capivasertib plus fulvestrant versus placebo plus fulvestrant in patients with aromatase inhibitor-resistant ER-positive, HER2-negative advanced breast cancer, including those who have received previous CDK4/6 inhibitors.

The primary analysis of FAKTION found that the addition of capivasertib to fulvestrant led to a progression-free survival improvement in the intention-to-treat population, with no apparent difference in benefit between the originally identified pathway-altered and pathway non-altered subgroups.^17^ However, the primary analysis identified PI3K/AKT/PTEN pathway-altered tumours using tests that had suboptimal sensitivity (pyrosequencing) or were unable to identify all activating *PIK3CA* mutations (ddPCR)*.* Moreover, PTEN abnormalities had been detected by protein loss rather than by the presence of genomic alterations, and tumours had not been examined for *AKT1* mutations. Correspondingly, only 59 (42%) of 140 participant tumours had been identified as PI3K/AKT/PTEN pathway altered,^17^ which is lower than the proportion detected by other studies.^7^, ^9^, ^11^ In this updated analysis, we did a prespecified exploratory subgroup analysis that used an expanded genetic testing panel to identify participants with PI3K/AKT/PTEN pathway-altered tumours with increased accuracy. The expanded pathway-altered subgroup accounted for 76 (54%) of the 140 participants in the intention-to-treat population. The subsequent subgroup analysis found that the significant progression-free survival and overall survival benefit of capivasertib identified in the intention-to-treat population was also present in the expanded pathway-altered subgroup, although not in the expanded pathway non-altered subgroup. We hypothesise, therefore, that the primary analysis^17^ failed to detect the increased capivasertib benefit for participants with PI3K/AKT/PTEN pathway-altered tumours because limitations of the original tests erroneously placed some participants with bone fide pathway-altered tumours in the pathway non-altered subgroup.

An additional post-hoc exploratory analysis suggested that NGS testing is needed to accurately identify patients who might not benefit from capivasertib, which was most apparent when considering progression-free survival results for patients identified as pathway non-altered using the different testing methods. Capivasertib treatment showed a significant progression-free survival benefit in the original pathway non-altered subgroup identified using ddPCR or pyrosequencing (or both), which was reduced to no significant difference in the expanded pathway non-altered subgroup that considered NGS results, and the HR approached 1 when pathway non-altered status was defined by NGS alone. Additionally, it is notable that the majority of tumour tissue samples were taken from the primary rather than the advanced tumour. In some cases, biopsies were taken many years before the participant entered the FAKTION trial, but the original tumour *PIK3CA, AKT1*, and *PTEN* status appeared to define the efficacy of capivasertib for advanced breast cancer.

The expanded pathway non-altered subgroup analysis suggests, but does not prove, that capivasertib predominantly benefits patients with PI3K/AKT/PTEN pathway alterations. Caution is required given that FAKTION studied a relatively small patient population and 20% of tumours were not tested by NGS. Additionally, several phase 1, 2, and 3 trials have shown that the relationship between tumour molecular profile and response to AKT inhibitors varies in ways that are as yet unclear and is probably related to both the context of the tumour and the combination drug partner. For example, the phase 2 ProCAID trial in participants with metastatic castration-resistant prostate cancer identified that the addition of capivasertib to docetaxel chemotherapy improved overall survival in patients with PI3K/AKT/PTEN pathway-altered and pathway non-altered tumours,^30^ and a phase 3 trial of another AKT inhibitor (ipatasertib) combined with abiraterone found efficacy in participants with PTEN-deficient metastatic castration-resistant prostate cancer and no treatment effect in the remainder of the study population.^31^ The varied use of different sample types as well as technologies including immunohistochemistry, ddPCR, and NGS assays could at least partially explain why it has been challenging to identify the tumour molecular profile that predicts the response to capivasertib. It is also not clear whether genomic analyses detect all forms of PI3K/AKT/PTEN pathway activation. Clear answers will require large phase 3 studies, and results from the CAPItello-291 trial in which NGS is used to identify *PIK3CA, AKT1,* and *PTEN* tumour alterations are eagerly awaited.

## Data sharing

Any requests for anonymised trial data or supporting material will be reviewed on a case-by-case basis. Only requests that have scientifically and methodologically sound proposals will be considered and the use of the shared trial data or supporting material will be limited to the approved proposal. The final decision as to whether data or supporting material might be shared and the exact data or supporting material to be shared will be made between the sponsor, trial team, and AstraZeneca. Proposals should be directed to the corresponding author.

## Declaration of interests

SJH received grant support for this study from AstraZeneca; research grants from Eli Lilly; consulting fees from Eli Lilly and Pfizer; honoraria from Pfizer; and participates on a trial steering committee for AstraZeneca. AC received grant support for the study from AstraZeneca and Cancer Research UK. RB received funding from the All Wales Medical Genetics Service (as head of the service) to do the original biomarker testing. PB reports honoraria from AstraZeneca and support from Eli Lilly related to meeting attendance and advisory or safety board participation. SMoo reports support for attending meetings from Eli Lilly and honoraria from AstraZeneca unrelated to the submitted work. CT received funding from AstraZeneca for the costs of doing this trial, as well as consulting fees and honoraria from AstraZeneca unrelated to the submitted work. SW declares honoraria from Novartis, consulting fees from Sanofi and Novartis, and travel expenses from Eli Lilly. ECdeB, GS, and AF are employees of and hold shares in AstraZeneca. AF receives funding from AstraZeneca to attend congresses and funding for capivasertib studies. RHJ reports grants from AstraZeneca and Cancer Research UK, and personal fees from Roche unrelated to the submitted work. All other authors declare no competing interests.

## References

[1] Howlader N, Altekruse SF, Li CI (2014). US incidence of breast cancer subtypes defined by joint hormone receptor and HER2 status. J Natl Cancer Inst.

[2] Cardoso F, Paluch-Shimon S, Senkus E (2020). 5th ESO-ESMO international consensus guidelines for advanced breast cancer (ABC 5). Ann Oncol.

[3] Brown JS, Banerji U (2017). Maximising the potential of AKT inhibitors as anti-cancer treatments. Pharmacol Ther.

[4] Coleman N, Moyers JT, Harbery A, Vivanco I, Yap TA (2021). Clinical development of AKT inhibitors and associated predictive biomarkers to guide patient treatment in cancer medicine. Pharm Genomics Pers Med.

[5] Baselga J, Im SA, Iwata H (2017). Buparlisib plus fulvestrant versus placebo plus fulvestrant in postmenopausal, hormone receptor-positive, HER2-negative, advanced breast cancer (BELLE-2): a randomised, double-blind, placebo-controlled, phase 3 trial. Lancet Oncol.

[6] Hortobagyi GN, Chen D, Piccart M (2016). Correlative analysis of genetic alterations and everolimus benefit in hormone receptor-positive, human epidermal growth factor receptor 2-negative advanced breast cancer: results from BOLERO-2. J Clin Oncol.

[7] Cancer Genome Atlas Network (2012). Comprehensive molecular portraits of human breast tumours. Nature.

[8] Stemke-Hale K, Gonzalez-Angulo AM, Lluch A (2008). An integrative genomic and proteomic analysis of *PIK3CA, PTEN,* and *AKT* mutations in breast cancer. Cancer Res.

[9] Turner NC, Kingston B, Kilburn LS (2020). Circulating tumour DNA analysis to direct therapy in advanced breast cancer (plasmaMATCH): a multicentre, multicohort, phase 2a, platform trial. Lancet Oncol.

[10] Bertucci F, Ng CKY, Patsouris A (2019). Genomic characterization of metastatic breast cancers. Nature.

[11] Razavi P, Chang MT, Xu G (2018). The genomic landscape of endocrine-resistant advanced breast cancers. Cancer Cell.

[12] André F, Ciruelos E, Rubovszky G (2019). Alpelisib for *PIK3CA*-mutated, hormone receptor-positive advanced breast cancer. N Engl J Med.

[13] André F, Ciruelos EM, Juric D (2021). Alpelisib plus fulvestrant for *PIK3CA*-mutated, hormone receptor-positive, human epidermal growth factor receptor-2-negative advanced breast cancer: final overall survival results from SOLAR-1. Ann Oncol.

[14] Baselga J, Campone M, Piccart M (2012). Everolimus in postmenopausal hormone-receptor-positive advanced breast cancer. N Engl J Med.

[15] Piccart M, Hortobagyi GN, Campone M (2014). Everolimus plus exemestane for hormone-receptor-positive, human epidermal growth factor receptor-2-negative advanced breast cancer: overall survival results from BOLERO-2†. Ann Oncol.

[16] Davies BR, Greenwood H, Dudley P (2012). Preclinical pharmacology of AZD5363, an inhibitor of AKT: pharmacodynamics, antitumor activity, and correlation of monotherapy activity with genetic background. Mol Cancer Ther.

[17] Jones RH, Casbard A, Carucci M (2020). Fulvestrant plus capivasertib versus placebo after relapse or progression on an aromatase inhibitor in metastatic, oestrogen receptor-positive breast cancer (FAKTION): a multicentre, randomised, controlled, phase 2 trial. Lancet Oncol.

[18] Schmid P, Abraham J, Chan S (2020). Capivasertib plus paclitaxel versus placebo plus paclitaxel as first-line therapy for metastatic triple-negative breast cancer: the PAKT trial. J Clin Oncol.

[19] Dent R, Oliveira M, Isakoff SJ (2021). Final results of the double-blind placebo-controlled randomized phase 2 LOTUS trial of first-line ipatasertib plus paclitaxel for inoperable locally advanced/metastatic triple-negative breast cancer. Breast Cancer Res Treat.

[20] Turner N, Dent RA, O'Shaughnessy J (2022). Ipatasertib plus paclitaxel for *PIK3CA*/*AKT1*/*PTEN*-altered hormone receptor-positive HER2-negative advanced breast cancer: primary results from cohort B of the IPATunity130 randomized phase 3 trial. Breast Cancer Res Treat.

[21] Pocock SJ, Simon R (1975). Sequential treatment assignment with balancing for prognostic factors in the controlled clinical trial. Biometrics.

[22] Smyth LM, Tamura K, Oliveira M (2020). Capivasertib, an AKT kinase inhibitor, as monotherapy or in combination with fulvestrant in patients with *AKT1* (E17K)-mutant, ER-positive metastatic breast cancer. Clin Cancer Res.

[23] Ng PK, Li J, Jeong KJ (2018). Systematic functional annotation of somatic mutations in cancer. Cancer Cell.

[24] Jin N, Keam B, Cho J (2021). Therapeutic implications of activating noncanonical *PIK3CA* mutations in head and neck squamous cell carcinoma. J Clin Invest.

[25] Cook MM, Al Rabadi L, Kaempf AJ, Saraceni MM, Savin MA, Mitri ZI (2021). Everolimus plus exemestane treatment in patients with metastatic hormone receptor-positive breast cancer previously treated with CDK4/6 inhibitor therapy. Oncologist.

[26] Turner NC, Slamon DJ, Ro J (2018). Overall survival with palbociclib and fulvestrant in advanced breast cancer. N Engl J Med.

[27] Rugo HS, Lerebours F, Ciruelos E (2021). Alpelisib plus fulvestrant in *PIK3CA*-mutated, hormone receptor-positive advanced breast cancer after a CDK4/6 inhibitor (BYLieve): one cohort of a phase 2, multicentre, open-label, non-comparative study. Lancet Oncol.

[28] Costa C, Wang Y, Ly A (2020). PTEN loss mediates clinical cross-resistance to CDK4/6 and PI3Kα inhibitors in breast cancer. Cancer Discov.

[29] Wander SA, Cohen O, Gong X (2020). The genomic landscape of intrinsic and acquired resistance to cyclin-dependent kinase 4/6 inhibitors in patients with hormone receptor-positive metastatic breast cancer. Cancer Discov.

[30] Crabb SJ, Griffiths G, Marwood E (2021). Pan-AKT inhibitor capivasertib with docetaxel and prednisolone in metastatic castration-resistant prostate cancer: a randomized, placebo-controlled phase II trial (ProCAID). J Clin Oncol.

[31] Sweeney C, Bracarda S, Sternberg CN (2021). Ipatasertib plus abiraterone and prednisolone in metastatic castration-resistant prostate cancer (IPATential150): a multicentre, randomised, double-blind, phase 3 trial. Lancet.

