# Supplementary material for: Fulvestrant plus capivasertib versus placebo after relapse or progression on an aromatase inhibitor in metastatic, oestrogen receptor-positive, HER2-negative breast cancer (FAKTION): overall survival, updated progression-free survival, and expanded biomarker analysis from a randomised, phase 2 trial
Source: Lancet Oncol. 2022 Jul;23(7):851–64. doi: 10.1016/S1470-2045(22)00284-4 (PMC9630162; doi:10.1016/S1470-2045(22)00284-4)
Supplement: Supplementary appendix [file mmc1.pdf]

THE LANCET
Oncology

Supplementary appendix

This appendix formed part of the original submission and has been peer reviewed. We post it as supplied by the authors.

Supplement to: Howell SJ, Casbard A, Carucci M, et al. Fulvestrant plus capivasertib versus placebo after relapse or progression on an aromatase inhibitor in metastatic, oestrogen receptor-positive, HER2-negative breast cancer (FAKTION): overall survival, updated progression-free survival, and expanded biomarker analysis from a randomised, phase 2 trial. *Lancet Oncol* 2022; published online June 4. https://doi.org/10.1016/S1470-2045(22)00284-4.

Supplemental Table 1: **FAKTION recruiting centres**

Site	PI	Number of participants registered	Number of participants randomised
The Christie NHS Foundation Trust, Manchester	Dr Sacha Howell	33	32
Velindre Cancer Centre, Velindre University NHS Trust, Cardiff	Dr Simon Waters	26	26
The Leeds Teaching Hospitals NHS Trust, St James's University Hospital, Leeds	Prof Chris Twelves	16	16
Calderdale and Huddersfield NHS Foundation Trust, Huddersfield	Prof Jonathan Joffe	12	11
University Hospitals of Morecambe Bay NHS Foundation Trust, Lancaster	Dr Sarah Moon	8	8
Betsi Cadwaladr University Health Board, Ysbyty Gwynedd, Bangor	Dr Catherine Bale	6	6
The Ipswich Hospital NHS Trust	Dr Ramachandran Venkitaraman	6	6
Blackpool Teaching Hospitals NHS Foundation Trust, Blackpool Victoria Hospital, Blackpool	Dr Pavel Bezecny	6	5
Barking, Havering and Redbridge University Hospitals NHS Trust, Queen’s Hospital, Romford	Dr Mary Quigley	4	4
Wrightington, Wigan and Leigh NHS Foundation Trust, Royal Albert and Edward Infirmary, Wigan	Dr Elena Takeuchi	4	4
Betsi Cadwaladr University Health Board, Glan Clwyd Hospital, Bodelwyddan, Rhyl	Dr Jill Bishop	4	4
The Clatterbridge Cancer Centre NHS Foundation Trust, Clatterbridge Cancer Centre, Liverpool	Prof Carlo Palmieri	4	4
Plymouth Hospitals NHS Trust, Derriford Hospital, Derriford	Dr Sidharth Dubey	4	4
University Hospitals Southampton NHS Foundation Trust, Southampton General Hospital, Southampton	Dr Ellen Copson	4	3
Lancashire Teaching Hospitals NHS Foundation Trust, Royal Preston Hospital, Preston	Dr Elaine Young	3	3
Royal Free London NHS Foundation Trust, Royal Free Hospital, London	Dr Jackie Newby	2	2
East Lancashire Hospitals NHS Trust, Royal Blackburn Hospital, Blackburn	Dr Martin Hogg	1	1
County Durham and Darlington NHS Foundation Trust, University Hospital North Durham, Durham	Dr Wendy Taylor	1	1
University Hospitals of North Midlands NHS Trust, Royal Stoke University Hospital, Stoke-on-Trent	Prof Murray Brunt	1	0

***Supplemental Figure 1*: Concordance between ddPCR/pyrosequencing, next-generation sequencing and immunohistochemical identification of PI3K/AKT/PTEN pathway altered and non-altered tumours**

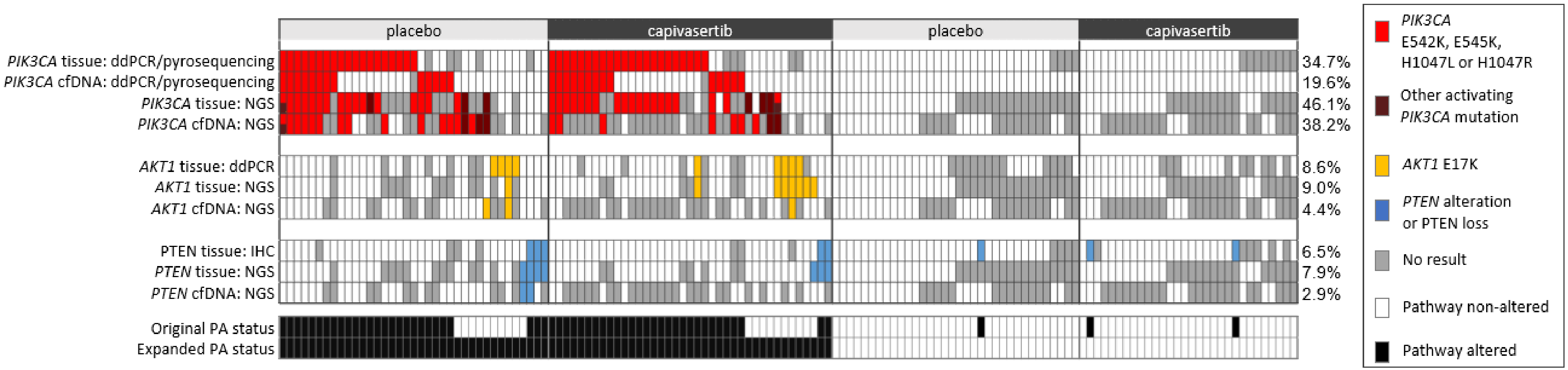

Tumour testing results and subgroup assignments of individual participants are arranged as columns, with each box indicating the result of a specific test (upper panel) or subgroup assignment (lower panel). Upper Panel: Red/burgundy, amber and blue boxes indicate that the assay identified a *PIK3CA*, *AKT1* or *PTEN* alteration (or PTEN loss), respectively. Multiple colours reflect that a tumour carried two types of *PIK3CA* alteration. White boxes indicate that the test did not detect an alteration. Grey boxes indicate that there was no test result, either because no additional tumour biopsy or plasma sample was available, or there was a test failure. Percentages (right) show how frequently each test (when it returned a result) identified each genetic alteration or PTEN deficiency. Lower panel: Participant subgroup assignments based on the original testing results and the expanded testing results. Black boxes indicate a participant was in the pathway altered subgroup and white boxes indicate that a participant was in the pathway non-altered subgroup.

cfDNA=cell free DNA; ddPCR=digital droplet polymerase chain reaction; IHC=immunohistochemistry; NGS=next-generation sequencing; tissue=tumour biopsy

Supplemental Table 2: **Proportional hazards assumption tests**

Comparison for analysis	N	Schoenfeld's test p-value
OS ITT	140	0·46
OS expanded pathway altered	76	0·23
OS expanded pathway non-altered	64	0·61
PFS ITT	140	0·77
PFS expanded pathway altered	76	0·75
PFS expanded pathway non-altered	64	0·71

ITT=intention-to-treat population; OS=overall survival; PFS=progression-free survival

Supplemental Table 3: **Baseline characteristics of the intention-to-treat population**

	Fulvestrant plus capivasertib (n=69)	Fulvestrant plus placebo (n=71)
**Age, years, median (IQR); range**	62·0 (55·0–68·0); 42–81	61·0 (53·0–68·0); 40–82
**Eastern Cooperative Oncology Group performance status (physical examination)**		
0	42 (61%)	49 (69%)
1	25 (36%)	17 (24%)
2	1 (1%)	2 (3%)
Missing	1 (1%)	3 (4%)
**Histopathological subtype**		
Invasive ductal carcinoma	57 (83%)	58 (82%)
Invasive lobular cancer	4 (6%)	12 (17%)
Mixed invasive ductal carcinoma and invasive lobular cancer	5 (7%)	0
Other	3 (4%)	1 (1%)
**Stage**		
III inoperable	0	1 (1%)
IV	68 (99%)	68 (96%)
Missing	1 (1%)	2 (3%)
**Number of disease sites**		
Median (IQR); range	2·0 (2·0–3·0); 1–5	2·0 (1·0–3·0); 1–5
1	13 (19%)	19 (27%)
2	56 (81%)	52 (73%)
**Metastatic sites***		
Brain	1 (1%)	1 (1%)
Liver	32 (46%)	29 (41%)
Lung	30 (43%)	28 (39%)
Bone	59 (86%)	55 (77%)
Lymph	28 (41%)	31 (44%)
Pericardial or pleural	5 (7%)	3 (4%)
Chest wall or skin	1 (1%)	3 (4%)
Other visceral	2 (3%)	1 (1%)
**Visceral disease**	49 (71%)	47 (66%)
**Measurable disease†**	49 (71%)	50 (70%)
**Primary or secondary aromatase inhibitor resistance†**		
Primary	25 (36%)	26 (37%)
Secondary	44 (64%)	45 (63%)
**Previous breast surgery**	59 (86%)	62 (87%)
**Previous adjuvant endocrine therapy**	60 (87%)	65 (92%)
Any tamoxifen	41 (59%)	45 (63%)
Any aromatase inhibitor	40 (58%)	38 (53%)
Any gonadotropin-releasing hormone	3 (4%)	1 (1%)
Other	1 (1%)	1 (1%)
Missing	0	1 (1%)
**Previous adjuvant chemotherapy**	36 (52%)	42 (59%)
Anthracycline based	11 (16%)	13 (18%)
Taxane based	5 (7%)	5 (7%)
Anthracycline plus taxane	11 (16%)	9 (13%)
Cyclophosphamide, methotrexate, and fluorouracil or capecitabine	7 (10%)	14 (20%)
Other	1 (1%)	1 (1%)
Missing	1 (1%)	0
**Previous endocrine treatment (metastatic or locally advanced setting)**		
0 lines	9 (13%)	6 (8%)
1 line	39 (57%)	45 (63%)
≥2 lines	20 (29%)	20 (28%)
Missing	1 (1%)	0
**Metastatic chemotherapy for advanced breast cancer**	17 (25%)	20 (28%)
Capecitabine based	3 (4%)	6 (8%)
Taxane based	8 (12%)	8 (11%)
Anthracycline based	2 (3%)	6 (8%)
Combined anthracycline and taxane	3 (4%)	0
Other	1 (1%)	0

Data are n (%), unless otherwise specified. The displayed percentages include missing values. *Sites are not mutually exclusive. †Randomisation minimisation factor.

IQR=interquartile range.

Supplemental Table 4: **Adverse events**

	Fulvestrant plus capivasertib (n=69) n (%)			Fulvestrant plus placebo (n=71) n (%)		
CTCAE grade	Grade 1/2	Grade 3	Grade 4	Grade 1/2	Grade 3	Grade 4
Hypertension (in patient study record)	41 (59%)	22 (32%)	0	45 (63%)	18 (25%)	0
Fatigue	40 (58%)	1 (1%)	0	39 (55%)	3 (4%)	0
Diarrhoea	47 (68%)	10 (14%)	0	22 (31%)	2 (3%)	1 (1%)
Nausea	39 (57%)	0	0	36 (51%)	0	0
Hypertriglyceridaemia (lab)	35 (51%)	2 (3%)	0	22 (31%)	0	0
Rash	23 (33%)	14 (20%)	0	14 (19%)	0	0
Pain (other)	23 (33%)	4 (6%)	0	18 (25%)	3 (4%)	0
Arthralgia	20 (29%)	2 (3%)	0	23 (32%)	0	0
ECG QT corrected interval prolonged	24 (35%)	0	0	19 (27%)	0	0
Cholesterol high (lab)	25 (36%)	0	0	18 (25%)	0	0
Injection site reactions	19 (28%)	1 (1%)	0	23 (32%)	0	0
Vomiting	26 (38%)	2 (3%)	0	15 (21%)	0	0
Elevated alkaline phosphatase (lab)	19 (28%)	1 (1%)	0	21 (30%)	1 (1%)	0
Infection (all)	24 (35%)	3 (4%)	1 (1%)	10 (14%)	2 (3%)	0
Urinary tract infection[δ]	11 (16%)	1 (1%)	0	5 (7%)	0	0
Respiratory tract infection[δ]	8 (12%)	2 (3%)	0	3 (4%)	1 (1%)	0
Headache	18 (26%)	0	0	23 (32%)	0	0
Hyperglycaemia (fasting)*	27 (39%)	3 (4%)	0	9 (13%)	0	0
Elevated alkaline phosphatase	16 (23%)	1 (1%)	0	17 (24%)	2 (3%)	0
Proteinuria (lab)	26 (38%)	0	0	8 (11%)	0	0
Haemoglobin decreased (lab)	19 (28%)	0	0	13 (18%)	1 (1%)	0
Hyperglycaemia (random timing) (lab)	23 (33%)	2 (3%)	0	7 (10%)	0	0
Elevated alanine transaminase	19 (28%)	0	0	12 (17%)	2 (3%)	0
Blood bilirubin decreased (lab)	18 (26%)	0	1 (1%)	11 (15%)	0	0
Back pain	15 (22%)	0	0	10 (14%)	2 (3%)	0
High-density lipoprotein (lab)	15 (22%)	0	0	12 (17%)	0	0
Low white blood cell count (lab)	14 (20%)	0	0	11 (15%)	0	0
Elevated alanine transaminase (lab)	14 (20%)	0	0	10 (14%)	1 (1%)	0
Low-density lipoprotein (lab)	14 (20%)	0	0	10 (14%)	0	0
Elevated aspartate aminotransferase	9 (13%)	0	0	10 (14%)	2 (3%)	0
Hot flashes	10 (14%)	0	0	12 (17%)	0	0
Urea (high) (lab)	14 (20%)	0	0	7 (10%)	0	0
Pain in extremity	10 (14%)	0	0	8 (11%)	1 (1%)	0
Hypoalbuminemia (lab)	13 (19%)	0	0	7 (10%)	0	0
Dyspnoea	6 (9%)	0	0	11 (15%)	1 (1%)	1 (1%)
Neutrophils (low) (lab)	10 (14%)	0	0	9 (13%)	0	0
Flu-like symptoms	9 (13%)	0	0	8 (11%)	0	0
Elevated aspartate aminotransferase (lab)	11 (16%)	0	0	7 (10%)	0	0
Creatinine increased (lab)	13 (19%)	0	0	5 (7%)	0	0
Abdominal pain	10 (14%)	1 (1%)	0	5 (7%)	1 (1%)	0

	Fulvestrant plus capivasertib (n=69) n (%)			Fulvestrant plus placebo (n=71) n (%)		
CTCAE grade	Grade 1/2	Grade 3	Grade 4	Grade 1/2	Grade 3	Grade 4
Mucositis oral	11 (16%)	0	0	5 (7%)	0	0
Non-cardiac chest pain	9 (13%)	0	0	6 (8%)	0	0
Cough	8 (12%)	0	0	7 (10%)	0	0
Constipation	6 (9%)	0	0	9 (13%)	0	0
Pulse (high) (in patient study record)	6 (9%)	0	0	9 (13%)	0	0
Neutrophils (high) (lab)	3 (4%)	1 (1%)	0	3 (4%)	0	0
Platelet count decreased	2 (3%)	0	1 (1%)	4 (6%)	1 (1%)	0
Anaemia	5 (7%)	0	0	0	2 (3%)	0
High white blood cell count (lab)†	4 (6%)	1 (1%)	0	2 (3%)	0	0
Sciatica	2 (3%)	1 (1%)	0	3 (4%)	0	0
Pleural effusion	2 (3%)	1 (1%)	0	2 (3%)	0	0
Acute kidney injury	3 (4%)	1 (1%)	0	1 (1%)	0	0
Peripheral neuropathy	2 (3%)	1 (1%)	0	2 (3%)	0	0
Fever	3 (4%)	0	0	0	1 (1%)	0
Hyponatraemia	1 (1%)	2 (3%)	0	0	1 (1%)	0
Hypoxia	0	1 (1%)	0	3 (4%)	0	0
Osteonecrosis of jaw	2 (3%)	1 (1%)	0	0	0	0
Gamma-glutamyl transferase increased	0	1 (1%)	0	1 (1%)	0	0
Blurred vision	0	1 (1%)	0	1 (1%)	0	0
Fracture	0	0	0	1 (1%)	1 (1%)	0
Syncope	1 (1%)	0	0	0	1 (1%)	0
Haemorrhage[#]	0	0	0	0	0	0
Blocked nephrostomy	0	1 (1%)	0	0	0	0
Eczema	0	1 (1%)	0	0	0	0
Muscle weakness lower limb	0	1 (1%)	0	0	0	0
Perineal abscess	0	1 (1%)	0	0	0	0
Pneumonia	0	1 (1%)	0	0	0	0
Ascites	0	0	0	0	1 (1%)	0
Gastroenteritis	0	0	0	0	1 (1%)	0
Joint swelling	0	0	0	0	1 (1%)	0

The table presents toxicities reported in at least 10% of patients in the intention-to-treat population or any toxicity reported at CTCAE grade 3 or higher, irrespective of cause. Categories that include "(in patient study record)" reflect that an abnormal reading was reported as an AE even if the investigator did not consider it as clinically significant. The CTCAE threshold for grade one hypertension is systolic blood pressure of 120–139 mm Hg or diastolic blood pressure of 80–89 mm Hg. Hypertension was recorded in 115 patients at entry to FAKTION before participants received study treatment. Categories that include the "(lab)" designation were toxicities reported from abnormal blood/biochemistry results that may not have had clinical significance. δ some participants had both urinary tract and respiratory tract infections. * includes patient-reported and laboratory-determined fasting values. † values were wrongly coded as low white blood cell counts in the primary analysis. [#] One patient in the treatment group had a grade 5 haemorrhage. Adverse events were classified according to CTCAE version 4.03.

CTCAE=Common Terminology Criteria for Adverse Events; ECG=electrocardiogram.

Results from expanded genetic testing

Expanded PI3K/AKT/PTEN pathway biomarker testing was performed on remaining participant tissue and/or plasma samples to detect the activating E17K *AKT1* mutation, additional *PIK3CA* mutations, and inactivating *PTEN* alterations, including that next-generation sequencing (NGS) results were obtained for 112 participant tumours. When considering the frequency that the expanded biomarker panel identified alterations in individual PI3K/AKT/PTEN pathway components, 43% (61 participants) of the intention-to-treat (ITT) population had tumours with at least one activating *PIK3CA* mutation, 8.6% (12 participants) carried *AKT1* E17K, and 5.0% (7 participants) had an inactivating *PTEN* alteration or *PTEN* gene loss. The expanded genetic testing identified *PIK3CA, AKT1* and/or *PTEN* alterations in tumours from 25% of participants originally considered as PI3K/AKT/PTEN pathway non-altered (figure 3). In total, the expanded testing found that 76 participants (of the N=140 ITT population) had PI3K/AKT/PTEN pathway altered tumours (the expanded pathway altered subgroup; 54% of the ITT population) as compared with 59 participants identified by the original tests (the original pathway altered subgroup; 42% of the ITT population). For participants whose tumour status was revised from pathway non-altered to pathway altered, eight had tumours with activating *PIK3CA* mutations, five of which were mutations that were not included in the original classification criteria (figure 3). There were an additional eight participants whose tumours carried the previously untested *AKT1* E17K mutation, one participant with a tumour carrying an inactivating *PTEN* alteration, and three with *AKT1* E17K in combination with either a *PIK3CA* mutation or a *PTEN* alteration. Three participants who had been assigned to the original pathway altered subgroup only on the basis of PTEN immunohistochemistry data were now categorised as pathway non-altered because NGS either did not detect a *PTEN* alteration or could not be performed for their tumours (figure 3).

***Supplemental Table 5*: Interaction tests**

The expanded testing panel tested pathway activation status in the intention-to-treat population. NGS refers to the subgroup of 112 patients where an NGS test determined pathway activation status. It is acknowledged there was low power in the study to test for interaction and no adjustment was made for multiplicity of testing.

Group	N	P value
overall survival intention-to-treat population	140	0·065
progression-free survival intention-to-treat population	140	0·18
overall survival NGS-tested	112	0·13
progression-free survival NGS-tested	112	0·046

NGS=next-generation sequencing

Supplemental Figure 2: **Progression-free survival and overall survival of participants with tumour PI3K/AKT/PTEN pathway altered status identified by next-generation sequencing of tissue and/or plasma samples**

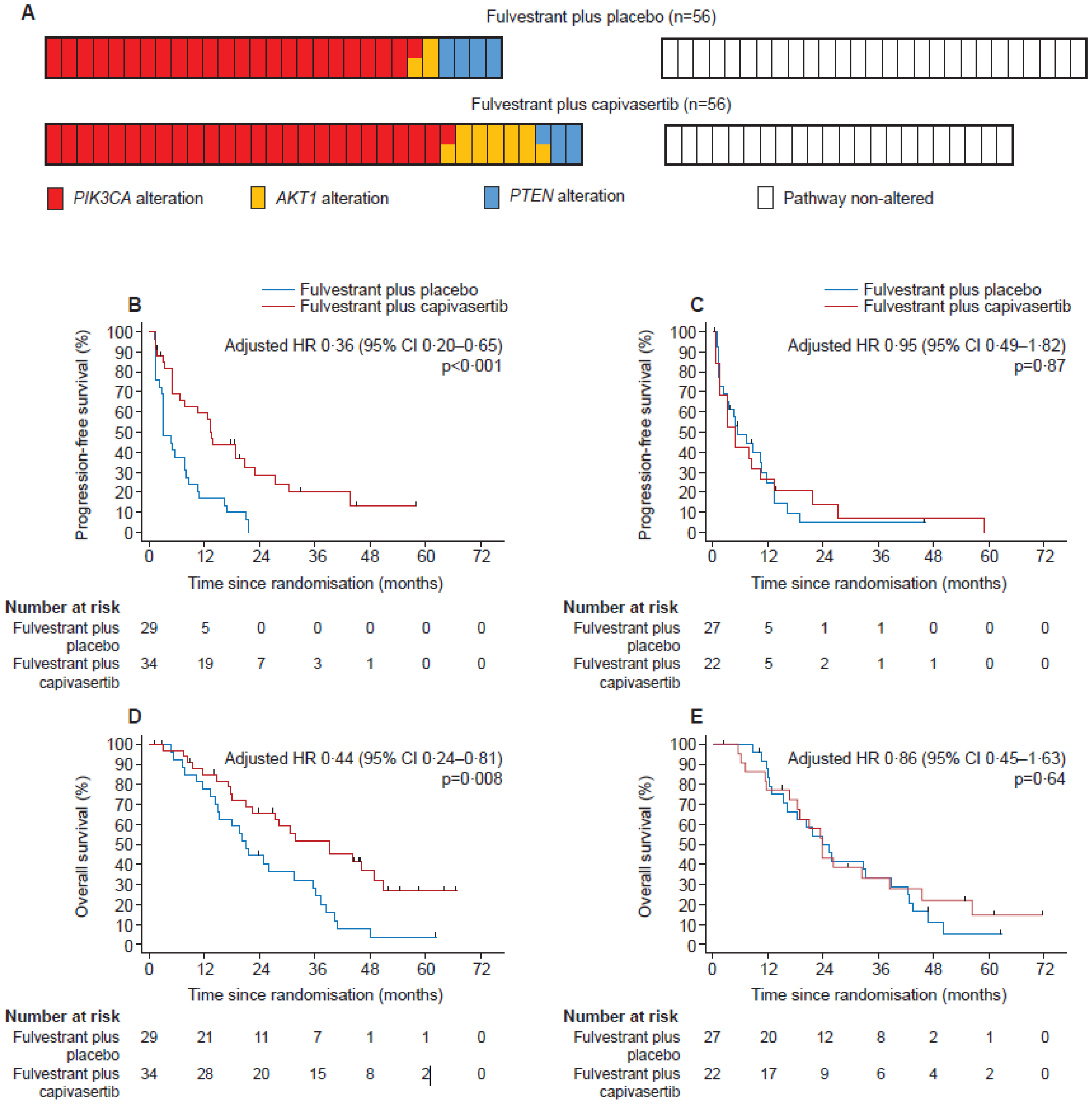

(A) Participants with PI3K/AKT/PTEN pathway altered tumour status identified by Foundation One® CDx (F1CDx) Clinical Trial NGS Assay testing of tumour biopsy samples and/or GuardantOMNI RUO Assay testing of plasma. Participants were considered to have a pathway altered tumour if either assay detected a *PIK3CA*, *AKT1*, or *PTEN* alteration. (B) Progression-free survival in the NGS-identified pathway altered subgroup. (C) Progression-free survival in the NGS-identified pathway non-altered subgroup. (D) Overall survival in the NGS-identified pathway altered subgroup. (E) Overall survival in the NGS-identified pathway non-altered subgroup. Tick marks show censoring events.

CI=confidence interval; HR=hazard ratio; NGS=next-generation sequencing.

Secondary Statistical Analysis Plan for FAKTION trial

[redacted]	[redacted]	[redacted]	[redacted]

Short Title: Fulvestrant +/- AKT inhibition in advanced aromatase inhibitor resistant breast cancer

Full Title: A phase 1b/2 randomised placebo controlled trial of fulvestrant +/- AZD5363 in postmenopausal women with advanced breast cancer previously treated with a third generation aromatase inhibitor

<u>Final Plan</u>

[redacted]

SAP Revision History

Protocol version	Updated Sap version no.	Section number changed	Description and reason for change	Date changed

ROLES AND RESPONSIBILITIES

**Trial Statistician:** [redacted]			
[redacted]			
[redacted]		Signature:	
**Senior Statistician:** [redacted]			
[redacted]			
[redacted]		Signature:	
**Chief Investigator:** [redacted]			
[redacted]			
[redacted]		Signature:	
**Chief Investigator:** [redacted]			
[redacted]			
[redacted]		Signature:	

TABLE OF CONTENTS

Table of Contents....3
1. INTRODUCTION....6
2. BACKGROUND....6
2.1 RATIONALE AND RESEARCH QUESTION....6
2.2 OBJECTIVES....7
RESEARCH HYPOTHESIS....7
2.2.1 STUDY OBJECTIVES....8
3. STUDY MATERIALS....8
3.1 TRIAL DESIGN....8
3.2 RANDOMISATION....8
3.3 BLINDING....9
3.4 SAMPLE SIZE....9
3.5 FRAMEWORK....9
3.6 INTERIM ANALYSES....9
3.6.1 PLANNED SAMPLE SIZE ADJUSTMENT....9
3.6.2 STOPPING RULES....9
3.7 TIMING OF FINAL ANALYSIS....9
3.8 PRE-ANALYSIS QUALITY CONTROL....9
4. STATISTICAL PRINCIPLES....10
5. STUDY POPULATION....11
5.1 CONCORDANCE....11
5.2 RULES FOR DEIVING SUBGROUPDS FOR ANALYSIS FOR THE PRIMARY ANALYSIS....12
6. ANALYSIS....12
6.1 OUTCOME DEFINITIONS....12
6.1.1 PRIMARY OUTCOME(S)....12
6.1.2 TIMING, UNITS AND DERIVATION OF PRIMARY....13
6.1.3 LIST OF SECONADRY OUTCOMES....13
6.1.4 ORDER OF TESTING....13
6.1.5 TIMING, UNITS AND DERVATION OF SECONDARYS....13
6.2 ANALYSIS METHODS....14
6.2.1 LIST OF METHODS AND PRESENTATION....14
6.2.2 COVARIATE ADJUSTMENT....14

6.2.3 ASSUMPTION CHECKING 15
6.2.4 ALTERNATIVE METHODS IF DISTRIBTIONAL ASSUMPTIONS NOT MET 15
6.2.5 SENSITIVITY ANALYSES 15
6.2.6 SUBGROUP ANALYSES 15
6.3 MISSING DATA 16
6.4 ADDITIONAL ANALYSES 16
6.5 HARMS....................16
6.6 STATISTICAL SOFTWARE 16
7. REFERENCES 16
7.1 NON STANDARD STATISTICAL METHODS....................16
7.2 DATA MANAGEMENT PLAN 16
7.3 TRIAL MASTER FILE AND STATISTICAL MASTER FILE....................16
7.4 OTHER SOPS OR GUIDANCE DOCUMENTS....................16
8. APPENDICES 17
Appendix 1a – Baseline mutational results by arm dummy table 17
Appendix 1b – Tissue Concordance dummy table, samples tested both at Cardiff and FMI only 19
Appendix 1c – Plasma Concordance dummy table, samples tested both at Cardiff and GH only 20
Appendix 2 – Flow chart to define inclusion criteria of each dataset....................21
Appendix 3 – Scenario 1....................22
Appendix 4 – Scenario 2....................22
Appendix 5 – Scenario 3....................23
Appendix 6 – Scenario 4....................23
Appendix 7 – Cardiff data only analyses....................24
Appendix 8 – FMI only analysis – all genes and per gene 24
Appendix 9 – tissue and plasma (if concordance is at least 90% for both tissue and for plasma) per gene 25
Appendix 10 – tissue and plasma per gene (if concordance is at least 90% for tissue, but less than 90% for plasma – exclude Cardiff plasma)....................25
Appendix 11: Progression Free Survival (PFS) and Overall Survival (OS) (ITT population)....................26
Appendix 12: Progression free survival (PFS) and overall survival (OS) by PIK3CA/PTEN pathway activation status (ITT population)....................27

Appendix 13: Kaplan-Meier curves of PFS by treatment arm (ITT population) 28

Appendix 14: Kaplan-Meier curves of OS by treatment arm (ITT population)..............28

Appendix 15: Kaplan-Meier curves of PFS stratified according to the PIK3CA/PTEN pathway activation status (ITT population)..28

Appendix 16: Kaplan-Meier curves of OS according to the PIK3CA/PTEN pathway activation status (ITT population)..28

1. INTRODUCTION

This statistical analysis plan (SAP) provides guidelines for the presentation of the secondary analysis for the FAKTION trial, which will assess mature overall survival data and explore whether different subgroup combinations of PI3K/AKT/PTEN genomic pathway alterations can identify patients most likely to respond to treatment. This plan, along with all other documents relating to the analysis of this trial, will be stored in the Statistical Analysis Master File electronically and/or in hard signed copy formats.

2. BACKGROUND

2.1 RATIONALE AND RESEARCH QUESTION

Breast cancer cells which have the estrogen receptor (ER positive or ER+) are more likely to grow when estrogen is present in the bloodstream. This can be treated by drugs that interfere with the action of estrogen or the estrogen receptor. Examples of this include tamoxifen, and the aromatase inhibitors (anastrozole letrozole exemestane and fulvestrant). Although these drugs are often effective for a while, the cancer can frequently become resistant, and the drugs stop working. Patients then require treatment with chemotherapy. In this trial, we are investigating whether, in patients with advanced aromatase inhibitor resistant breast cancer, we can reverse resistance to hormone therapy by adding an additional oral drug called capivasertib (formerly known as AZD5363). This is a targeted therapy that blocks the action of a cellular protein called AKT, which has been shown to cause resistance to hormone therapy. We will combine this drug with fulvestrant, another hormone therapy which is sometimes used alone in patients who have developed resistance to Aromatase Inhibitors, or Tamoxifen. Thus, patients entering the trial will have a known drug with proven efficacy and will also possibly receive the experimental drug which may enhance activity.

Value and Impact using Tumour Samples for genetic analysis at Foundation Medicine Inc.

The FAKTION trial assessed the efficacy of Capivasertib (AZD5363) in combination with fulvestrant vs fulvestrant plus placebo in terms of progression free survival in women with ER+ advanced/metastatic breast cancer (MBC). A key secondary outcome was to assess the influence of mutational status of PIK3CA and the level of expression of PTEN on outcome in the two treatment groups. To identify the mutational status of PIK3CA, the FAKTION study team used an ddPCR assay covering four common hotspot mutations in the PIK3CA gene, and an IHC assay to identify absence of PTEN protein expression.

The FAKTION study showed a benefit of capivasertib in combination with fulvestrant in the overall population, and also showed that the PI3K pathway alteration status as defined above did not seem to impact the effect size of capivasertib (Jones et al., *Lancet Oncol* 2020). However, AKT1 and non-hotspot PIK3CA mutation status (anticipate ~7% AKT1 mutant and ~5% non-hotspot PIK3CA mutant in ER+ MBC) and PTEN mutations were not tested at the time of this publication and, therefore, included in the non-altered subgroup. Thus, it remains

unanswered as to whether or not a broader approach to selecting relevant pathway alterations identifies a population that is more likely to respond to the combination of fulvestrant and capivasertib. Understanding this by ensuring that all aspects of pathway alteration are characterised versus the clinical outcomes in FAKTION will inform the statistical analysis plans of the now ongoing Phase 3 study CAPItello-291 which is recruiting rapidly.

In agreement with Sponsor and Funder, the available plasma samples, and formalin-fixed paraffin-embedded tumour tissue samples from consenting FAKTION participants have been re-tested respectively by Guardant Health and Foundation Medicine Inc. with a validated Next-Generation Sequencing (NGS) test covering all gene alterations of interest.

The samples analysis with this platform, which has significant technical advantages over the ddPCR assay, will enable a more robust assessment of the benefit of capivasertib in combination with fulvestrant in patients whose tumours harbour an eligible PIK3CA/AKT1/PTEN alteration.

We will also investigate whether DNA alterations or loss of protein in the original cancer specimen or on cancer cells in the blood determine the likelihood of treatment success. This will be possible by examining the original cancer specimen from a previous biopsy or operation, and a blood test when entering the trial.

This SAP outlines the analysis for the exploratory biomarkers including next generation sequencing (NGS) analysis of PIK3CA and AKT1 alterations, and PTEN deletions. This was outlined as a future analysis with a separate SAP in the original FAKTION SAP.

2.2 OBJECTIVES

RESEARCH HYPOTHESIS

The null hypothesis for the Overall Survival (OS) analysis is that there is no difference in OS between patients who were treated with fulvestrant plus capivasertib and those treated with fulvestrant alone.

The null hypothesis for the Progression Free Survival (PFS) analysis is that there is no difference in PFS between patients who were treated with fulvestrant plus capivasertib and those treated with fulvestrant alone.

The null hypothesis for the mutational analysis is that there are no differences in OS and PFS between those participants within genomic pathway alteration sub-groups who were treated with fulvestrant plus capivasertib and those treated with fulvestrant alone.

The alternative hypotheses are that there are differences between the two groups.

2.2.1 STUDY OBJECTIVES

1. The primary objective is to investigate OS, defined as the time from randomisation to death with those still alive censored at date of last assessment.
2. The secondary objective is to investigate PFS, defined as the time from randomisation to any disease progression and/or any death, defined according to RECIST v1.1 criteria.
3. The third objective is to identify whether PI3K/AKT/PTEN pathway activation alterations predict benefit to capivasertib.

3. STUDY MATERIALS

3.1 TRIAL DESIGN

This is a four-stage study, with an initial dose escalation phase 1b study (stage 1) which is now closed, and a subsequent double blind randomised phase 2 controlled trial (stages 2-4), which is currently in follow up. The phase 2 study is divided into three stages as pre-clinical data support the concept that capivasertib may be more active in tumours with PI3K/AKT/PTEN pathway activation. From each enrolled participant one archival tumour tissue was collected at screening and two 10ml plasma samples were collected at screening, Cycle 3, and End of Treatment. The trial is designed to test this concept in the clinical setting, with a pre-planned interim analysis, whilst minimising exposure of capivasertib in patients potentially less likely to benefit from it.

3.2 RANDOMISATION

After consent, participants were randomised through logging in to the Interactive Web Response System (IWRS) for the trial on 1:1 participant treatment allocation ratio with the main aim to reduce bias. The method of minimization ensured balance between treatment arms by using the following four clinical important stratification variables:

- PIK3CA alteration status (wildtype or mutated)
- PTEN level (0/1+ in <10% of tumour cells versus >1+ or 1+ in ≥10% tumour cells)
- Measurable versus non-measurable disease
- Resistance to prior AI therapy (Primary or secondary resistance)
    - Primary resistance is defined as either 1) disease relapse during or within 6 months (i.e., 26 weeks) of completing AI treatment in the adjuvant setting, or 2) disease progression within 6 months of starting AI treatment and no response to AI treatment in the metastatic setting
    - Secondary resistance is defined as 1) disease relapse more than 6 months (i.e., 26 weeks) after completion of AI treatment in the adjuvant setting, or 2) disease progression following achievement of clinical benefit with AI therapy to treat MBC.

3.3 BLINDING

This is a double-blind trial. All patients received fulvestrant in an unblinded manner, but both patients and clinicians (or research investigators) were blinded to the capivasertib or oral placebo.

3.4 SAMPLE SIZE

The original sample size was calculated for a Phase 2 screening design, based on a primary outcome of PFS, assuming a time-to-event hazard ratio of 0.65, 90% power, a one-sided significance of 20% and assuming an overall loss to follow-up of 10%. Assuming that the estimated PFS in the control arm is 5.4 months, and that we will have an 18-month accrual period and a 6-month follow-up, then a total of 98 events will be required overall. We aimed to recruit 138 patients in order to detect 98 progression events. A total of 140 patients were randomised.

3.5 FRAMEWORK

This is a superiority trial, based on the research hypothesis that the addition of capivasertib to fulvestrant will be more efficacious than fulvestrant alone. This has been demonstrated by an increase in PFS (using RECIST 1.1) in patients with ER+ve advanced or metastatic breast cancer.

It is also hypothesized that breast cancers that have activation of tumour PI3K/AKT/PTEN signalling will have increased sensitivity to capivasertib and, therefore, demonstrate greater improvement in PFS to capivasertib combined with fulvestrant than those with fulvestrant alone.

3.6 INTERIM ANALYSES

N/A

3.6.1 PLANNED SAMPLE SIZE ADJUSTMENT

N/A

3.6.2 STOPPING RULES

N/A

3.7 TIMING OF FINAL ANALYSIS

Analysis of OS will take place when we have at least 98 death events, and analysis of full PI3K/AKT/PTEN will take place when we receive the NGS data back from Foundation Medicine and Guardant Health. We will lock the clinical database for this analysis when we have confirmation of the 98 death events.

3.8 PRE-ANALYSIS QUALITY CONTROL

Internal validation is set up within the MACRO database to check that patient identifiers on each form are consistent with the original details given at registration. Internal date validation includes checks that dates are consistent and the time differences between dates are within

plausible ranges. Range checks are performed on all continuous data. Cross checks include those for missing or inconsistent data related to a previous qualifier. Cross-checks will be made during data cleaning and will include in particular:

- Check for missing CRFs
- Confirmation of ineligibility for any participants found to be ineligible after randomisation
- Confirmation of any patients with protocol violations that should be considered as affecting data integrity
- Check consistency of dates of withdrawal and death against CRF return
- Consistency of withdrawal data and progression data. Confirm those not evaluable for RECIST.
- Consistency in order of dates or long periods between dates
- Confirm consistency of tumour lesion data with reported RECIST response
- For patients that have progressed, check consistency of RECIST response data given at baseline and subsequent RECIST assessments
- Confirm disease progression and death events
- For patients that died, check data on cause of death is not missing, disease progression before death and dates of death are after any other dates reported in the CRFs

<u>All</u> annotated programs are signed off and kept in the FAKTION Statistical Analysis Master File. Other checks are detailed in the FAKTION Data Cleaning Plan.

4. STATISTICAL PRINCIPLES

4.1 LEVELS OF CONFIDENCE AND P-VALUES

Both one-sided and two-sided tests will be provided for the original PFS analysis, but two-sided p-values will be reported for all other analyses.

4.1.1 ADJUSTMENT FOR MULTIPLICITY

As these analyses are secondary to the main analysis, analysis 1 (Appendix 3-6) will be the primary subgroup for analysis, and interaction p-values will be assessed. All other subgroup analyses are treated as exploratory and no adjustment for multiplicity will be made.

See section 6.2.6 on subgroup definitions.

4.2 ADHERENCE AND PROTOCOL DEVIATIONS

4.2.1 DEFINITION AND ASSESSSMENT OF ADHERENCE

N/A

4.2.2 PRESENTATION OF ADHERENCE

N/A

4.2.3 DEFINITION OF PROTOCOL DEVIATION

N/A

4.2.4 PRESENTATION OF PROTOCOL DEVIATIONS

N/A

4.3 ANALYSIS POPULATION

The data will be analysed from four different assays:

1. archival tumour tissue samples analysed by Cellular Pathology and All Wales Genetics Laboratory, University Hospital of Wales = Cardiff tissue;
2. baseline blood plasma samples analysed by All Wales Genetics Laboratory, University Hospital of Wales = Cardiff plasma;
3. archival tumour tissue samples analysed by FMI = FMI;
4. blood plasma samples analysed by Guardant Health = GH.

Analysis sets will depend on the availability of pathway activation results (Appendix 1a), whether NGS data are available and whether Cardiff data are available (Appendix 2, and Appendix 3 to Appendix 6).

If the patient has an overall missing result according to Table 1 below, they will be excluded from the analysis.

5. STUDY POPULATION

We will describe numbers screened, eligible, withdrawals in an updated consort diagram.

5.1 CONCORDANCE

As the data are being analysed from four different assays, there is an expectation that some of the positive results may differ across the four sources.

For PIK3CA and AKT1 genes combined: Where both a Cardiff and FMI tissue were tested and at least 90% of mutations identified in Cardiff are also identified by FMI, the Cardiff tissue data will be deemed reliable, and mutations accepted where no FMI sample was tested. If greater than 10% mutations detected in Cardiff are not confirmed by FMI (effectively false positives in Cardiff, if we assume FMI to be the 'gold standard'), the Cardiff plasma data will be excluded from the primary subgroup (Analyses 1 to 3 – Appendix 3 to Appendix 6).

For the plasma PIK3CA data only 64 patients had a baseline plasma sample analysed by GH. It is thus most important that we do not unnecessarily exclude mutant calls from Cardiff in the absence of a GH result. Where both a Cardiff and GH plasma sample were tested and at least 90% of mutations identified in Cardiff are also identified by GH then Cardiff plasma data will be deemed reliable, and mutations accepted where no GH sample was tested. If greater than 10% of Cardiff mutations are not confirmed by GH (effectively false positives in Cardiff if we assume GH to be the 'gold standard') then the Cardiff plasma data will be excluded from the primary subgroup (Analyses 1 to 3 – Appendix 3 to Appendix 6).

A flowchart has been included in Appendix 2 to outline these scenarios. Each possible combination of datasets has been outlined in Appendix 3 to Appendix 6. One table will demonstrate the final analyses depending on the scenario based on levels of concordance between the Cardiff/FMI tissue, Cardiff/GH plasma for PIK3CA and Cardiff/FMI tissue for AKT1 (Appendix 2).

5.2 RULES FOR DEIVING SUBGROUPDS FOR ANALYSIS FOR THE PRIMARY ANALYSIS

Depending on which datasets will be included in the final analyses (defined above), the following rules will be used to derive the subgroups and final categorisations of the PIK3CA and AKT1 data:

1. Mutant on FMI = mutant, irrespective of all other results
2. Mutant on GH = mutant, irrespective of all other results

If there is at least 90% concordance between PIK3CA and AKT1 mutant data in Cardiff tissue and FMI, use Cardiff tissue where no FMI data is available:

3. Accept mutant in Cardiff tissue (even if GH is negative for a mutant)
4. Accept AKT1 in Cardiff tissue

If there is at least 90% concordance between PIK3CA and AKT1 mutant data in Cardiff plasma and GH, use Cardiff plasma where no GH data is available:

5. Accept Cardiff plasma mutant even if Cardiff tissue and FMI are negative

- Negative result if all of the above are negative, see table below for unknown/missing guidance.

Table 1 - Rules for missing values and unknowns in the analyses

PIK3CA	AKT1	PTEN	Final status
Negative	Negative	Missing/unknown	Negative
Negative	Missing/unknown	Negative	Negative
Negative	Missing/unknown	Missing/unknown	Negative
Missing/unknown	Negative	Negative	Missing
Missing/unknown	Negative	Missing/unknown	Missing
Missing/unknown	Missing/unknown	Negative	Missing

6. ANALYSIS

6.1 OUTCOME DEFINITIONS

6.1.1 PRIMARY OUTCOME(S)

This is a secondary analysis of the primary outcome PFS and secondary outcome OS, in conjunction with our pre-defined subgroup for PI3K/AKT/PTEN pathway activation.

6.1.2 TIMING, UNITS AND DERIVATION OF PRIMARY

OS will be measured as the time from randomisation to death with those still alive censored at date last assessed.

6.1.3 LIST OF SECONADRY OUTCOMES

NA – no further analyses of secondary outcomes are planned.

6.1.4 ORDER OF TESTING

We will analyse different subgroups for PI3K/AKT/PTEN pathway activation in the order given in Appendices 3 to 11. Only one of Appendices 3 to 6 will be carried out depending on the level of concordance between Cardiff Tissue and FMI data, and Cardiff Plasma Data and GH data (defined in Appendix 2). The primary subgroup for this analysis will be analysis 1 in Appendix 3 to Appendix 6, depending on the level of concordance (Appendix 2).

6.1.5 TIMING, UNITS AND DERVATION OF SECONDARYS

PFS is defined as the time from randomisation to any disease progression and/or any death, defined according to strict RECIST v1.1 criteria.

The disease response is collected on paper based RECIST CRFs. The radiology report identifies the target lesions and non-target lesions that are to be followed for the trial (if the patient has measurable disease at baseline). The report includes measurements of the lesions (and describes any new lesions) and the research nurse transfers this information onto the RECIST CRF and manually assigns the response on the RECIST CRF.

- RECIST assessments will be performed at the end of: cycle 2; cycle 4; cycle 6; cycle 9; and then at the end of every 3 cycles until disease progression.
- The overall response variable will be used at every timepoint to determine whether the patient has complete response, partial response, stable disease, progressive disease, or non-evaluable disease.
- PFS is defined as the time from randomisation until the date of objective disease progression or death (by any cause in the absence of progression) regardless of whether the patient withdraws from study therapy or receives another anti-cancer therapy prior to progression (i.e., date of PFS event or censoring – date of first randomisation + 1). Patients who have not progressed or died at the time of analysis will be censored at the time of the latest date of assessment from their last evaluable RECIST assessment. However, if the patient progresses or dies after two or more missed RECIST assessments, the patient will be censored at the time of the latest evaluable RECIST 1.1 assessment prior to the two missed visits.
- If the patient has no evaluable visits or does not have baseline data, they will be censored at Day 1 post randomisation unless they die within 2 visits of baseline (16 weeks plus 1 week allowing for a late assessment within the visit window).
- The PFS time will always be derived based on scan/assessment dates, not visit dates.
- RECIST assessments/scans contributing towards a particular visit may be performed on different dates. The following rules will be applied:

- For investigational assessments, the date of progression will be determined based on the earliest of the dates of the component that triggered the progression
- When censoring a patient for PFS the patient will be censored at the latest of the dates contributing to a particular overall visit assessment

The outcome of time to progressive disease will be derived by determining whether the patient has had a progression or qualifying death event.

If the patient has had an event, data from the corresponding RECIST (or death) form is used to set the indicator variable for progression event to 1 and to set the follow-up time to the date of the RECIST assessment (or the date of death).

If the patient has not had an event, then the indicator variable for progression is set to 0, and the follow-up time is set to the date the patient last attended a trial visit (or the date of death if the death does not qualify as an event).

6.2 ANALYSIS METHODS

6.2.1 LIST OF METHODS AND PRESENTATION

Survival:

- We will calculate survival (PFS and OS) with PFS defined as time from randomisation to any disease progression and/or any death, defined according to strict RECIST v1.1 and OS defined as time from randomisation to death with those still alive censored at date last seen. Patients who were event free will be censored at the time they were last known to be event free (last RECIST assessment).
- We will estimate event time distributions with the Kaplan-Meier method and compare PFS and OS with an unadjusted log-rank test and HRs from Cox regression by trial arm for each subgroup in Appendix 13. Hazard ratios, CIs, and p values will be calculated for PFS using cox regression in univariable and multivariable models in Appendix 11.
- We will perform a subgroup analysis to estimate the HR for PFS and OS according to varying definitions of PIK3CA pathway activation status (Appendix 12), see section 7.2.5.
- We will test the proportional hazards assumption with Cox-Snell residuals and Schoenfeld's global test.
- Multivariable analyses will adjust for the randomisation stratification variables.

6.2.2 COVARIATE ADJUSTMENT

We will run sensitivity analyses for the ITT population and by activated pathway sub-populations, using multivariable models to adjust for relevant baseline patient characteristics including visceral disease, measurable disease, endocrine sensitivity, age, pathway activation, resistance to prior AI therapy (primary/secondary) and last prior therapy (AI, anti-estrogen), and any other baseline factors that appear unbalanced). Factors with stratas of <5 will be

pooled with the next strata if there are 3 or more strata or removed. Survival multivariable analyses will adjust for the randomisation stratification variables.

6.2.3 ASSUMPTION CHECKING

For survival endpoints, we will test the proportional hazards assumption with Cox-Snell residuals and Schoenfeld's global test.

6.2.4 ALTERNATIVE METHODS IF DISTRIBTIONAL ASSUMPTIONS NOT MET

If the assumptions are not met, then Cox regression using the stratification factors will not be performed and instead a comparison of restricted mean survival time will be employed.

6.2.5 SENSITIVITY ANALYSES

No formal sensitivity analyses are planned.

6.2.6 SUBGROUP ANALYSES

Subgroup analysis will be performed according to PI3K/AKT/PTEN pathway activation status.

The original definition of PI3K pathway activation (Cardiff results) is as follows:

PI3K/PTEN pathway activated: at least one alteration observed in one of the markers i.e.

PIK3CA gene alteration in either cfDNA or tumour block analysis and/or low/reduced expression of PTEN analysed by immunochemistry (< 10% of tumour cells expressing PTEN at no more than 1+ level) independent of availability of remaining test results.

PI3K /PTEN pathway non-activated (wildtype): no alterations detected in PIK3CA gene in cfDNA or tumour block analysis and PTEN expression detectable in ≥ 10% of tumour cells at 1+ or in any tumour cells at >1+. Where PIK3CA alteration is un-interpretable in cfDNA but no alteration is found in tumour block DNA analysis, such a participant's tumour will be classified as pathway non-activated.

PI3K /PTEN pathway unknown: at least 1 un-interpretable result from analyses of the tumour block only (alteration or IHC) combined with no criteria of activation as defined above in any of the markers; does not meet the definition of PI3K pathway activated or non-activated.

Note that for the purposes of defining PTEN in FAKTION samples, all of the samples found to express PTEN were positive in more than 10% tumour cells, so a PTEN score of 0 is counted as pathway activated and PTEN score of 1, 2, or 3 is counted as non-activated.

In the NGS analysis of tumour and plasma samples, alterations in PIK3CA, AKT1 and PTEN which met the threshold for significant alteration are identified in NGS results files as being Tier1 alterations.

Analysis sub-groups definitions for OS and PFS outcomes can be found in Appendix 3 to Appendix 10. Subgroup comparisons will be displayed in Kaplan-Meier curve in Appendix 15 and Appendix 16.

6.3 MISSING DATA

There is no planned data imputation for missing data in this study. Every effort will be made to collect the data by querying study sites. Additionally, every effort will be made to obtain data on patients lost to follow up through the NHSIC.

6.4 ADDITIONAL ANALYSES

There will be no additional analyses.

6.5 HARMS

The adverse reactions and toxicities were reported as part of the original SAP.

6.6 STATISTICAL SOFTWARE

The analysis will be carried out using Stata version 17.

7. REFERENCES

7.1 NON STANDARD STATISTICAL METHODS

7.2 DATA MANAGEMENT PLAN

7.3 TRIAL MASTER FILE AND STATISTICAL MASTER FILE

7.4 OTHER SOPS OR GUIDANCE DOCUMENTS

SAP/ISAP DEVIATION LOG

**Document number:**		**Document version:**	
**Reason for deviation:**			

8. APPENDICES

Appendix 1a – Baseline mutational results by arm dummy table

			Fulvestrant plus Placebo Arm (n=71)		Fulvestrant plus AZD5363 Arm (n=69)	
			N	%	N	%
**Cardiff**	**PIK3CA results: blood**	No alteration				
		Alteration				
		Missing				
	**PIK3CA results: tissue**	No alteration				
		Alteration				
		Missing				
	**PTEN IHC results**	0				
		1				
		2				
		3				
		Missing				
	**AKT1 results**	No alteration				
		Alteration				
		Missing				
**GH**	**PIK3CA**	No alteration				
		Alteration				
		Unknown				
		Missing				
	**AKT1**	No alteration				
		Alteration				

		Unknown				
		Missing				
	PTEN	No alteration				
		Alteration				
		Unknown				
		Missing				
**FMI**	**PIK3CA**	No alteration				
		Alteration				
		Unknown				
		Missing				
	**AKT1**	No alteration				
		Alteration				
		Unknown				
		Missing				
	**PTEN**	No alteration				
		Alteration				
		Unknown				
		Missing				

Appendix 1b – Tissue Concordance dummy table, samples tested both at Cardiff and FMI only

Number of patient tissue samples tested in both Cardiff and by FMI	N=		
	FMI PIK3CA or AKT mutation detected	FMI PIK3CA or AKT not detected	Total Cardiff
Cardiff Tissue PIK3CA or AKT mutation	n=A	n=B	n=E
Cardiff Tissue PIK3CA or AKT mutation not detected	n=C	n=D	n=F
Concordance %	=(A/E) x 100		

Appendix 1c – Plasma Concordance dummy table, samples tested both at Cardiff and GH only

Number of patient plasma samples tested in both Cardiff and by GH	N=		
	GH PIK3CA or AKT mutation detected	GH PIK3CA or AKT not detected	Total Cardiff
Cardiff Plasma PIK3CA or AKT mutation	n=A	n=B	n=E
Cardiff Plasma PIK3CA or AKT mutation not detected	n=C	n=D	n=F
Concordance %	=(A/E) x 100		

Appendix 2 – Flow chart to define inclusion criteria of each dataset

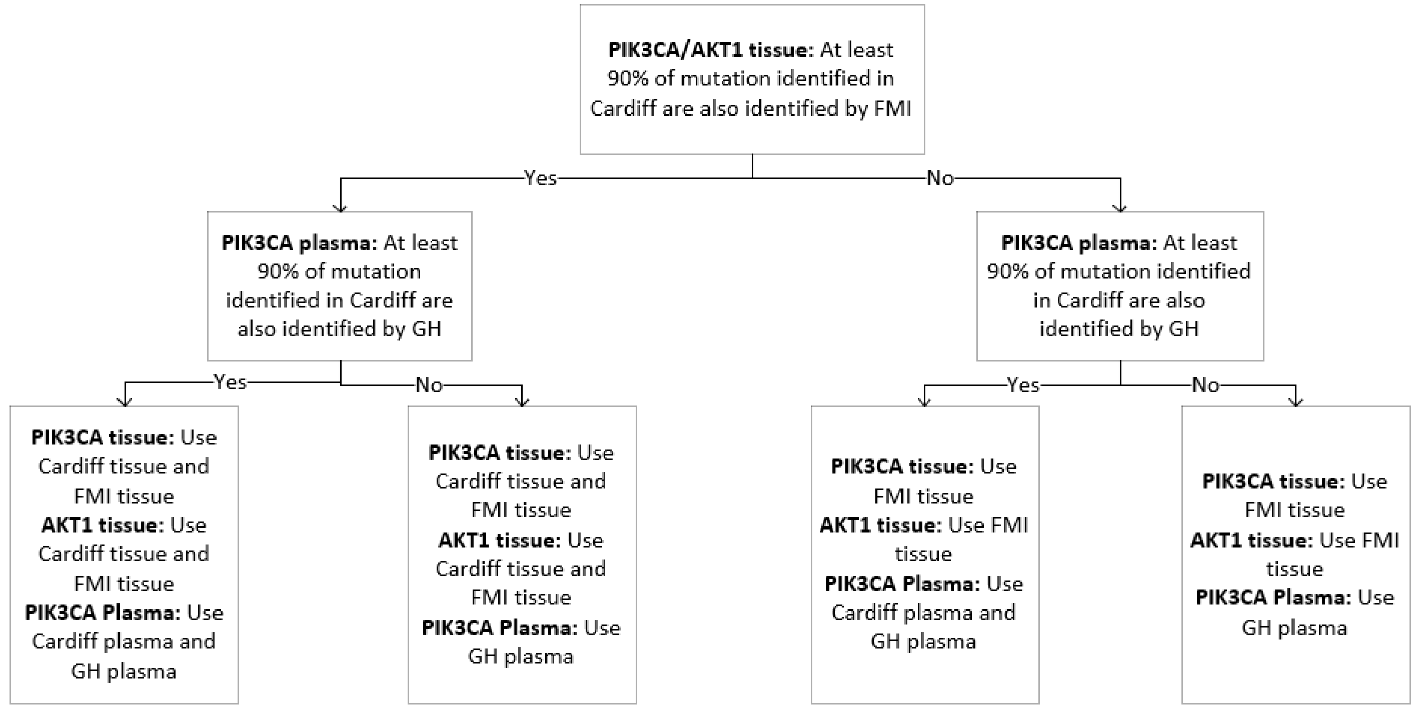

	Scenario 1	Scenario 2	Scenario 3	Scenario 4
PIK3CA Cardiff Tissue	✓	✓	☒	☒
PIK3CA FMI Tissue	✓	✓	✓	✓
AKT1 Cardiff Tissue	✓	✓	☒	☒
AKT1 FMI Tissue	✓	✓	✓	✓
PIK3CA Cardiff Plasma	✓	☒	✓	☒
PIK3CA GH Plasma	✓	✓	✓	✓

Appendix 3 – Scenario 1

**Gene**	**PIK3CA**				**AKT1**			**PTEN**		
**Method**	**Cardiff ddPCR**		**FMI NGS**	**GH NGS**	**Cardiff ddPCR**	**FMI NGS**	**GH NGS**	**IHC**	**FMI NGS**	**GH NGS**
**Source**	**Tissue (FFPE)**	**Plasma (ctDNA)**	**Tissue (FFPE)**	**Plasma (ctDNA)**	**Tissue (FFPE)**	**Tissue (FFPA)**	**Plasma (ctDNA)**	**Tissue (FFPE)**	**Tissue (FFPE)**	**Plasma (ctDNA)**
**Analysis 1 - all**	PIK3CA alteration	PIK3CA alteration	PIK3CA alteration	PIK3CA alteration	AKT1 alteration	AKT1 alteration	AKT1 alteration	-	PTEN alteration	PTEN alteration
**Analysis 2 - tissue**	PIK3CA alteration	-	PIK3CA alteration	-	AKT1 alteration	AKT1 alteration	-	-	PTEN alteration	-
**Analysis 3 - plasma**	-	PIK3CA alteration	-	PIK3CA alteration	-	-	AKT1 alteration		-	PTEN alteration

Appendix 4 – Scenario 2

**Gene**	**PIK3CA**				**AKT1**			**PTEN**		
**Method**	**Cardiff ddPCR**		**FMI NGS**	**GH NGS**	**Cardiff ddPCR**	**FMI NGS**	**GH NGS**	**IHC**	**FMI NGS**	**GH NGS**
**Source**	**Tissue (FFPE)**	**Plasma (ctDNA)**	**Tissue (FFPE)**	**Plasma (ctDNA)**	**Tissue (FFPE)**	**Tissue (FFPA)**	**Plasma (ctDNA)**	**Tissue (FFPE)**	**Tissue (FFPE)**	**Plasma (ctDNA)**
**Analysis 1 - all**	PIK3CA alteration	-	PIK3CA alteration	PIK3CA alteration	AKT1 alteration	AKT1 alteration	AKT1 alteration	-	PTEN alteration	PTEN alteration
**Analysis 2 - tissue**	PIK3CA alteration	-	PIK3CA alteration	-	AKT1 alteration	AKT1 alteration	-	-	PTEN alteration	-
**Analysis 3 - plasma**	-	-	-	PIK3CA alteration	AKT1 alteration	-	AKT1 alteration	-	-	PTEN alteration

Appendix 5 – Scenario 3

Gene	PIK3CA				AKT1			PTEN		
Method	Cardiff ddPCR		FMI NGS	GH NGS	Cardiff ddPCR	FMI NGS	GH NGS	IHC	FMI NGS	GH NGS
Source	Tissue (FFPE)	Plasma (ctDNA)	Tissue (FFPE)	Plasma (ctDNA)	Tissue (FFPE)	Tissue (FFPA)	Plasma (ctDNA)	Tissue (FFPE)	Tissue (FFPE)	Plasma (ctDNA)
**Analysis 1 - all**	-	PIK3CA alteration	PIK3CA alteration	PIK3CA alteration	-	AKT1 alteration	AKT1 alteration	-	PTEN alteration	PTEN alteration
**Analysis 2 - tissue**	-	-	PIK3CA alteration	-	-	AKT1 alteration	-	-	PTEN alteration	-
**Analysis 3 - plasma**	-	PIK3CA alteration	-	PIK3CA alteration	-	-	AKT1 alteration	-	-	PTEN alteration

Appendix 6 – Scenario 4

Gene	PIK3CA				AKT1			PTEN		
Method	Cardiff ddPCR		FMI NGS	GH NGS	Cardiff ddPCR	FMI NGS	GH NGS	IHC	FMI NGS	GH NGS
Source	Tissue (FFPE)	Plasma (ctDNA)	Tissue (FFPE)	Plasma (ctDNA)	Tissue (FFPE)	Tissue (FFPA)	Plasma (ctDNA)	Tissue (FFPE)	Tissue (FFPE)	Plasma (ctDNA)
**Analysis 1 - all**	-	-	PIK3CA alteration	PIK3CA alteration	-	AKT1 alteration	AKT1 alteration	-	PTEN alteration	PTEN alteration
**Analysis 2 - tissue**	-	-	PIK3CA alteration	-	-	AKT1 alteration	-	-	PTEN alteration	-
**Analysis 3 - plasma**	-	-	-	PIK3CA alteration	-	-	AKT1 alteration	-	-	PTEN alteration

Appendix 7 – Cardiff data only analyses

Gene	PIK3CA				AKT1			PTEN		
**Method**	**Cardiff ddPCR**		**FMI NGS**	**GH NGS**	**Cardiff ddPCR**	**FMI NGS**	**GH NGS**	**IHC**	**FMI NGS**	**GH NGS**
**Source**	**Tissue (FFPE)**	**Plasma (ctDNA)**	**Tissue (FFPE)**	**Plasma (ctDNA)**	**Tissue (FFPE)**	**Tissue (FFPA)**	**Plasma (ctDNA)**	**Tissue (FFPE)**	**Tissue (FFPE)**	**Plasma (ctDNA)**
**Analysis 4a**	PIK3CA alteration	PIK3CA alteration	-	-	-	-	-	PTEN alteration	-	-
**Analysis 4b**	PIK3CA alteration	PIK3CA alteration	-	-	AKT1 alteration	-	-	-	-	-
**Analysis 4c**	PIK3CA alteration	PIK3CA alteration	-	-	AKT1 alteration	-	-	PTEN alteration	-	-

Appendix 8 – FMI only analysis – all genes and per gene

Gene	PIK3CA				AKT1			PTEN		
**Method**	**Cardiff ddPCR**		**FMI NGS**	**GH NGS**	**Cardiff ddPCR**	**FMI NGS**	**GH NGS**	**IHC**	**FMI NGS**	**GH NGS**
**Source**	**Tissue (FFPE)**	**Plasma (ctDNA)**	**Tissue (FFPE)**	**Plasma (ctDNA)**	**Tissue (FFPE)**	**Tissue (FFPA)**	**Plasma (ctDNA)**	**Tissue (FFPE)**	**Tissue (FFPE)**	**Plasma (ctDNA)**
**Analysis 5a**	-	-	PIK3CA alteration	-	-	AKT1 alteration	-	-	PTEN alteration	-
**Analysis 5b**	-	-	PIK3CA alteration	-	-		-	-	-	-
**Analysis 5c**	-	-	-	-	-	AKT1 alteration	-	-	-	-
**Analysis 5d**	-	-	-	-	-	-	-	-	PTEN alteration	-

Appendix 9 – tissue and plasma (if concordance is at least 90% for both tissue and for plasma) per gene

Gene	PIK3CA				AKT1			PTEN		
Method	Cardiff ddPCR		FMI NGS	GH NGS	Cardiff ddPCR	FMI NGS	GH NGS	IHC	FMI NGS	GH NGS
Source	Tissue (FFPE)	Plasma (ctDNA)	Tissue (FFPE)	Plasma (ctDNA)	Tissue (FFPE)	Tissue (FFPA)	Plasma (ctDNA)	Tissue (FFPE)	Tissue (FFPE)	Plasma (ctDNA)
**Analysis 6a**	- PIK3CA alteration	PIK3CA alteration -	PIK3CA alteration	PIK3CA alteration -	-	-	-	-	-	-
**Analysis 6b**	-	-	-	-	AKT1 alteration	AKT1 alteration	AKT1 alteration	-	-	-
**Analysis 6c**	-	-	-	-	-	-	-	-	- PTEN alteration	- PTEN alteration

Appendix 10 – tissue and plasma per gene (if concordance is at least 90% for tissue, but less than 90% for plasma – exclude Cardiff plasma)

Gene	PIK3CA				AKT1			PTEN		
Method	Cardiff ddPCR		FMI NGS	GH NGS	Cardiff ddPCR	FMI NGS	GH NGS	IHC	FMI NGS	GH NGS
Source	Tissue (FFPE)	Plasma (ctDNA)	Tissue (FFPE)	Plasma (ctDNA)	Tissue (FFPE)	Tissue (FFPA)	Plasma (ctDNA)	Tissue (FFPE)	Tissue (FFPE)	Plasma (ctDNA)
**Analysis 7a**	- PIK3CA alteration	-	PIK3CA alteration	- PIK3CA alteration	-	-	-	-	-	-
**Analysis 7b**	-	-	-	-	AKT1 alteration	AKT1 alteration	AKT1 alteration	-	-	-
**Analysis 7c**	-	-	-	-	-	-	-	-	PTEN alteration	PTEN alteration

Appendix 11: Progression Free Survival (PFS) and Overall Survival (OS) (ITT population)

	**Fulvestrant plus Placebo arm (n=xx)**		**Fulvestrant plus capivasertib arm (n=xx)**	
Progression Free Survival (PFS)				
Total events, n (%)	xx	x%	xx	x%
RECIST progression	xx	x%	xx	x%
Death in the absence of progression	xx	x%	xx	x%
Censored patients, n (%)	xx	x%	xx	x%
Progression-free at time of analysis	xx	x%	xx	x%
Lost to follow-up	xx	x%	xx	x%
Withdrawn consent	xx	x%	xx	x%
**Median progression-free survival (months)**	xx		xx	
95% CI for median progression-free survival	xx.x - xx.x		xx.x - xx.x	
**Unadjusted log-rank test, 1-sided p-value**	0.xxx			
Hazard ratio, unadjusted	xx			
95% CI for hazard ratio	xx.x - xx.x			
2-sided p-value	xx			
Hazard ratio, adjusted for randomisation stratification factors*	xx			
95% CI for hazard ratio	xx.x - xx.x			
2-sided p-value	xx			
Hazard ratio, adjusted for randomisation stratification factors plus other covariates	xx			
95% CI for hazard ratio	xx.x - xx.x			
2-sided p-value	xx			
Overall Survival (OS)				
**Median overall survival (months)**	xx		xx	
95% CI for median overall survival	xx.x - xx.x		xx.x - xx.x	
Hazard ratio, unadjusted	xx			
95% CI for hazard ratio	xx.x - xx.x			
2-sided p-value	xx			
Hazard ratio, adjusted for randomisation stratification factors	xx			
95% CI for hazard ratio	xx.x - xx.x			
2-sided p-value	xx			
**Median duration of follow-up (months)**	x (xx.x - xx.x)		x (xx.x - xx.x)	

*Pathway activation status, Measurable versus non-measurable disease, Resistance to prior AI therapy (primary or secondary resistance)

Appendix 12: Progression free survival (PFS) and overall survival (OS) by PIK3CA/PTEN pathway activation status (ITT population)

	PI3K/PTEN Pathway activated (n=xx)		PI3K/PTEN Pathway non-activated (wildtype) or unknown (n=xx)	
	Fulvestrant plus Placebo arm (n=xx)	Fulvestrant plus capivasertib arm (n=xx)	Fulvestrant plus Placebo arm (n=xx)	Fulvestrant plus capivasertib arm (n=xx)
Progression Free Survival (PFS)				
Total events, n (%)	xx x%	xx x%	xx x%	xx x%
RECIST progression	xx x%	xx x%	xx x%	xx x%
Death in the absence of progression	xx x%	xx x%	xx x%	xx x%
Censored patients, n (%)	xx x%	xx x%	xx x%	xx x%
Progression-free at time of analysis	xx x%	xx x%	xx x%	xx x%
Lost to follow-up	xx x%	xx x%	xx x%	xx x%
Withdrawn consent	xx x%	xx x%	xx x%	xx x%
**Median progression-free survival (months)**	xx	xx	xx	xx
95% CI for median progression-free survival	xx.x - xx.x	xx.x - xx.x	xx.x - xx.x	xx.x - xx.x
Hazard ratio, unadjusted	xx		xx	
95% CI for hazard ratio	xx.x - xx.x		xx.x - xx.x	
2-sided p-value	xx		xx	
Hazard ratio, adjusted for randomisation stratification factors*	xx		xx	
95% CI for hazard ratio	xx.x - xx.x		xx.x - xx.x	
2-sided p-value	xx		xx	
Hazard ratio, adjusted for randomisation stratification factors plus additional covariates	xx		xx	
95% CI for hazard ratio	xx.x - xx.x		xx.x - xx.x	
2-sided p-value	xx		xx	
Overall Survival (OS)				
**Median overall survival (months)**	xx	xx	xx	xx
95% CI for median overall survival	xx.x - xx.x	xx.x - xx.x	xx.x - xx.x	xx.x - xx.x
Hazard ratio, unadjusted	xx		xx	
95% CI for hazard ratio	xx.x - xx.x		xx.x - xx.x	
2-sided p-value	xx		xx	
Hazard ratio, adjusted for randomisation stratification factors	xx		xx	
95% CI for hazard ratio	xx.x - xx.x		xx.x - xx.x	
2-sided p-value	xx		xx	
**Median duration of follow-up (months)**	x (xx.x - xx.x)	x (xx.x - xx.x)	x (xx.x - xx.x)	x (xx.x - xx.x)

Appendix 13: Kaplan-Meier curves of PFS by treatment arm (ITT population)

Kaplan-Meier survival curves of treatment arms measuring the time from randomization to any disease progression and/or any death, defined as according to RECIST v1.1.

Appendix 14: Kaplan-Meier curves of OS by treatment arm (ITT population)

Kaplan-Meier survival curves of treatment arms measuring the time from randomization to death from any causes.

Appendix 15: Kaplan-Meier curves of PFS stratified according to the PIK3CA/PTEN pathway activation status (ITT population)

Kaplan-Meier survival curves stratified according to the PIK3CA/PTEN pathway activation status measuring the time from randomization to any disease progression and/or any death, defined as according to RECIST v1.1.

Appendix 16: Kaplan-Meier curves of OS according to the PIK3CA/PTEN pathway activation status (ITT population)

Kaplan-Meier survival curves stratified according to the PIK3CA/PTEN pathway activation status measuring the time from randomization to death from any causes.
